# Spatio-Temporal Metabolite Profiling of the Barley Germination Process by MALDI MS Imaging

**DOI:** 10.1371/journal.pone.0150208

**Published:** 2016-03-03

**Authors:** Karin Gorzolka, Jan Kölling, Tim W. Nattkemper, Karsten Niehaus

**Affiliations:** 1 Proteome and Metabolome Research, Faculty of Biology, Center for Biotechnology (CeBiTec), Bielefeld, Germany; 2 Biodata Mining, Faculty of Technology, Center for Biotechnology (CeBiTec), Bielefeld, Germany; 3 International Research Training Group "Computational Methods for the Analysis of the Diversity and Dynamics of Genomes", Bielefeld University, Bielefeld, Germany; The University of Melbourne, AUSTRALIA

## Abstract

MALDI mass spectrometry imaging was performed to localize metabolites during the first seven days of the barley germination. Up to 100 mass signals were detected of which 85 signals were identified as 48 different metabolites with highly tissue-specific localizations. Oligosaccharides were observed in the endosperm and in parts of the developed embryo. Lipids in the endosperm co-localized in dependency on their fatty acid compositions with changes in the distributions of diacyl phosphatidylcholines during germination. 26 potentially antifungal hordatines were detected in the embryo with tissue-specific localizations of their glycosylated, hydroxylated, and O-methylated derivates. In order to reveal spatio-temporal patterns in local metabolite compositions, multiple MSI data sets from a time series were analyzed in one batch. This requires a new preprocessing strategy to achieve comparability between data sets as well as a new strategy for unsupervised clustering. The resulting spatial segmentation for each time point sample is visualized in an interactive cluster map and enables simultaneous interactive exploration of all time points. Using this new analysis approach and visualization tool germination-dependent developments of metabolite patterns with single MS position accuracy were discovered. This is the first study that presents metabolite profiling of a cereals’ germination process over time by MALDI MSI with the identification of a large number of peaks of agronomically and industrially important compounds such as oligosaccharides, lipids and antifungal agents. Their detailed localization as well as the MS cluster analyses for on-tissue metabolite profile mapping revealed important information for the understanding of the germination process, which is of high scientific interest.

## Introduction

Cereals are the basis of human nutrition. During the last 20,000 years of cultivation, crops underwent constant improvements in yield and quality. Among cereals, barley ranks fourth for worldwide production with 144 million tons in 2013 [1]. Besides its role as cattle feed, barley is of high cultural importance with 20% of the production being processed by malting, providing the basis for beer as well as for non-alcoholic drinks. Due to its nutritional benefits, there is an increasing interest in its use as functional food [2,3]. The germination rate and natural pathogen resistance are key factors for production yield and grain quality. Thus, the investigation and the understanding of the molecular events of cereal germination and pathogen defense will benefit agriculture and human nutrition.

Barley is a model organism for the investigation of the cereal germination process. The mature barley seed is composed of different tissues with distinct functions during germination. The dead endosperm serves for storage of starch, proteins, and other molecules to supply the embryo with nutrients during early germination. The outer cell layers of the endosperm build the aleurone layer. In contrast to the dead endosperm, the aleurone layer and the embryo are viable, but dormant in the mature seed. This dormancy is broken by the uptake of water, which induces the release of gibberellic acid (GA) from the embryo. GA promotes the production and release of hydrolytic enzymes from the aleurone layer into the inner endosperm. Endosperm components (mainly amylose, amylopectin, proteins, and cell wall components like arabinoxylan and beta glucan) are hydrolyzed, e.g. to oligo- and monosaccharides, peptides, and amino acids. A subset of these compounds is translocated through the scutellum to the embryo to provide energy and building blocks for its growth and respiration [4,5].

Several investigations addressed the understanding of the germination process covering protein analyses [6], transcriptomics [7], and metabolite profiling [8]. Germination is a complex scenario of molecular interactions between tissues, but also with functional compartmentalization. Common analytical methods include extraction steps with the loss of detailed information about compound localizations and resulting in complex molecular patterns. These problems were addressed by the dissection of seeds in main compartments (e.g. [6]) or using *in vitro* cultivation of cells and tissues (e.g. of the aleurone layer [4]). However, the separation of the dead inner endosperm, the aleurone layer, and embryo is a simplification of tissue complexity and the distinction of subfractions is desirable to examine tissue functionality during germination.

Mass spectrometry imaging (MSI) provides a platform for the detailed localization of diverse molecular species such as metabolites, lipids, peptides, and proteins in a sample section with spatial resolutions of micrometer scales [9–13]. MSI is well suited for targeted localization approaches (e.g. drug imaging [14]) as well as for untargeted profiling of tissue sections. Sample preparation and instrumental equipment determine the range of detectable compounds, with ionization methods (e.g. SIMS, DESI, LAESI, MALDI) and MS detectors (e.g. FT-ICR, Orbitrap, TOF MS) as the main parameters. In MALDI MSI, the chemistry of the matrix is relevant, as different matrices promote the ionization of molecules of certain chemical classes [9–13].

The localization of compounds as provided by MALDI MSI links the metabolites to potential functionality. Therefore, MSI is frequently applied in clinical, especially in cancer research to detect tissue- and disease-specific molecular patterns [15–18]. In comparison to the numerous studies of clinical background, MSI of plants remains sparse [13]. In plant MSI, small molecules were the main targeted substances, investigating different plant species like *Arabidopsis*, *Medicago*, wheat, soya, rice, and tobacco with tissues such as leaves, stems, roots, flowers or fruits [12,13,19]. The identification of plant metabolites often focused on specific classes such as phosphatidylcholines [20,21], cell wall constituents [22], anthocyanins [23] or other secondary metabolites as summarized in a comprehensive review by Bjarnholt et al. [12], Boughton et al. [13] and Matros and Mock [19],. Novel metabolites were discovered using MALDI MSI [15], demonstrating the benefit of localization information for metabolite identification. The analysis of ripe cereal grains by MSI is challenging, since the rigidity and friability of the seed hamper the required sectioning and mounting. Wheat and barley were investigated during maturation, either when grains were still unripe and soft [24] or after incubation of the ripe seed in water [22]. Rice was the sole mature cereal that was investigated by MSI without prior sample manipulation by incubation [20]. No other mature cereal and no cereal germination process were investigated by MSI until today. Considering the convincing analytical perspectives of MS imaging, its application on a cereal germination process would reveal a lot of novel information on metabolite localizations, abundances and metabolic compartmentalization at a glance. This needs novel protocols for seed sectioning, sample preparation and especially data processing as introduced in the later sections. Barley was chosen, since it exhibits typical cereal germination nature, contains typical germination-relevant metabolites such as sugars and lipids, and represents an industrially and agriculturally relevant crop. Therefore, the results of localization and the identifications of compounds in barley can be also expected for other cereals. Since the established analysis also provides a protocol for sectioning, sample preparation, data acquisition and interpretation, MSI can be easily performed on various other crops, diverse tissues or time scale analyses.

A joint analysis of spatial patterns across all time points was conducted. To achieve a comparative analysis of multiple full MSI data sets a protocol for data preprocessing and analysis was applied as presented in this work to address the specific problems raised by the data generated in our study.

The first problem to be solved is to choose an appropriate pre-processing protocol. In MSI, even a single 2D data set can reach a size that is prohibitive to many types of analyses–if applied naively–and spectra are known to have a high pixel-to-pixel variance caused by a multitude of technical and chemical sources of noise and variation [25–27]. While the analysis of single 2D MSI data sets is feasible with current commercial tools (e.g. SCiLS Lab (SCiLS GmbH, Germany), flexImaging (Bruker)) and open resources [28,29] many publications are still neglecting the importance of proper preprocessing or avoid in-depth statistical analysis, due to the difficulty of defining clear criteria for an efficient preprocessing.

The comparative analysis of multiple MSI data sets, for example from a time series experiment as considered in this work, poses additional challenges that require tailor-made processing and analysis. MSI applications that could be categorized as comparative are: (A) Identification of new, or search for known biomarkers, also called MSI profiling [30]; (B) 3D MSI based on serial-sectioning of the same sample and subsequent 2D MSI of each section [31,32]; (C) Comparative MSI of multiple MSI data sets of individual samples [33,34], which is considered in this work. The goal of computationally assisted comparative MSI is to enable exploration of complex relationships between spatial patterns and molecular composition across samples from multiple individuals, for example from different time points of a biological process. This requires a broader comparison than the targeted MSI profiling (see (A)) and has to account for inherently more biological variability than present in a single 3D MSI data set (see (B)). The major challenge is therefore to make all spectra of all data sets under consideration comparable under the noisy conditions of MALDI MSI and additional batch effects between samples [35]. This can be tackled by accounting for variability in exact *m/z* positions–usually through re-calibration of the *m/z* axis–and normalization of signal intensities. In absence of established quality measures or ground truth for most kinds of samples, prior knowledge on the analyzed data is essential to support and assess this automated processing.

The second problem is to find a way to analyze and visualize the data in a way that allows for intuitive interpretation of both spatial patterns and single molecular profiles. Both techniques for dimension reduction [36,37] as well as clustering algorithms [38,39] are commonly applied to distill relevant information from MSI data sets. The resulting lower dimensional embedding is frequently visualized in the context of optical images of the samples to enable researchers to use their knowledge of sample morphology during interpretation. To this end, the molecular composition is reduced to a color encoding clusters or regions described by special patterns in molecular composition [36] and is mapped to the optical image.

We present an approach to both the automated processing as well as the interactive visual exploration of the results in an open web tool. The usefulness of the approach is demonstrated by the in-depth spectral analysis of a single data set as well as by the joint spatio-temporal analysis of eight individual data sets obtained during barley seed germination. This analytical protocol together with the recorded data sets provided a fundamental new view on metabolite distributions during germination, as it allows a simultaneous analysis of temporal and spatial molecular organization. With the identification of 85 out of in total 101 detected metabolite signals, metabolite (co-)localizations as well as metabolite cluster results could be reasonably interpreted, which is of high interest for crop agriculture.

## Material and Methods

### Mini malting and sample collection

100 g barley seeds (cv. *Optic*, provided by the Campden Brewery Research Institute (BRI), Brewing Division, Nutfield, UK) were germinated in 1 l Schott flasks at 16°C in an air-conditioned dark cabinet. Seeds underwent cycles of soaking and rest periods with 7 h soaking in water, 17 h rest (after draining), 7 h soaking, 17 h rest (drained), 3 h soaking, 1 h draining, followed by 5 days of germination. After the five days of germination period, seeds were dried for 7 h at 45°C and kilned at 65°C for 17 h. This procedure represents the industrial malting process. During the soaking and germination period, samples were collected in 24 h steps, immediately frozen at −20°C on a plate, transferred in reaction tubes, and stored at −80°C.

### Sample preparation and MALDI-TOF MSI

**Fig 1:**
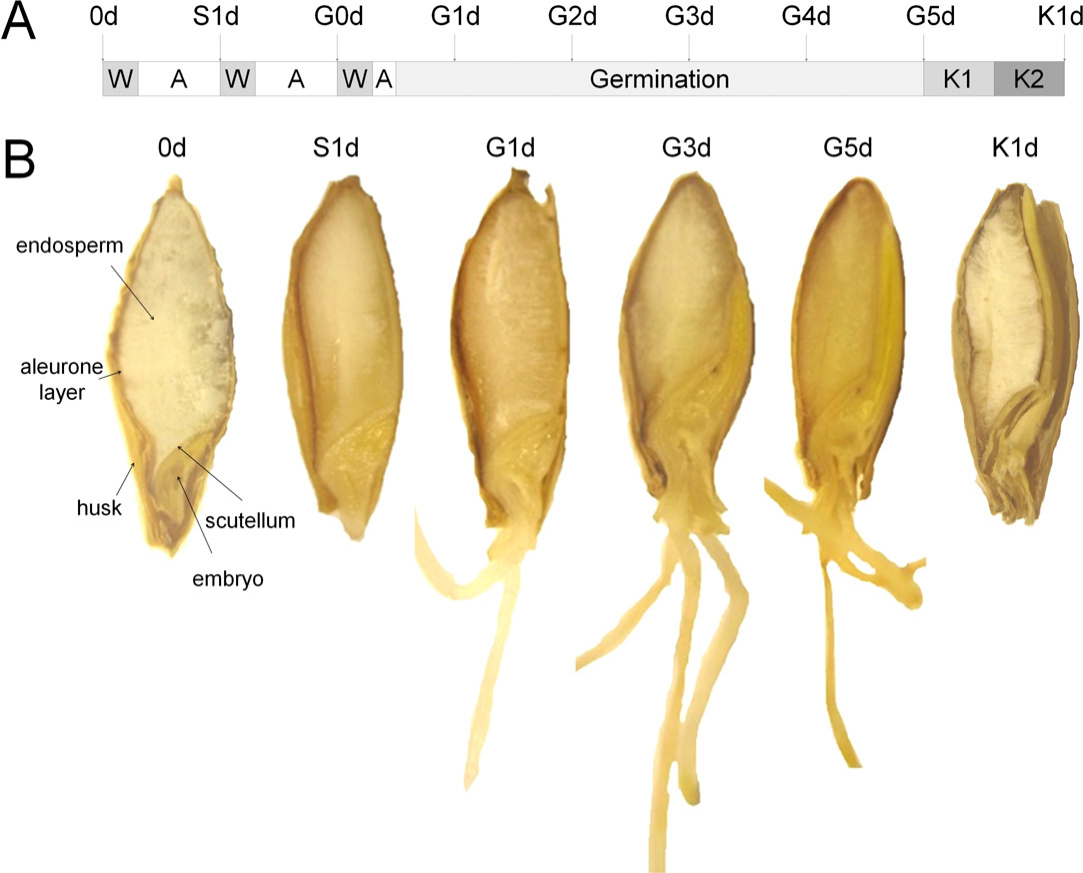
The barley germination process: Seedling development and sampling time points. A) Time scheme of mini malting of the *Optic* barley at 16°C for the collection of samples. Arrows indicate sampling time points with their sample name. 0d: raw barley, S: steeping, G: germination day, K: kilned malt (K not used for MSI). W: water, A: air rest, K1: kilning at 45°C (7h), K2: kilning at 65°C (17 h). B) Growth of the barley seeds during malting. Barley (0d, *T* = 1), steeped barley (S1d, *T* = 2), three of the five time points during germination (G1d, G3d, G5d (*T* = 4,6,8)) and final kilned malt (K1d) are shown as representatives. Main seed organs and compartments are indicated at the raw barley seed.

Samples of eight germination time points (Fig 1) were prepared and analyzed as described previously [15]. Briefly, seeds were embedded in ice and sectioned with 14 μm layer thickness at −20°C with a cryostat (Leica CM 1850) according to Kawamotos film method [20]. After immediate drying in a vacuum desiccator, samples on the film were mounted by electric conductive tape on an indium tin oxide (ITO)-coated conductive glass slide (Bruker Daltonics, Bremen, Germany), coated with matrix (30 mg/ml 2,5-dihydroxybenzoic acid (99%, Alfa Aesar) in 50% (v/v) methanol/water and 0.2% trifluoroacetic acid (Sigma Aldrich)) using the ImagePrep sprayer (Bruker Daltonics), and measured by MALDI-TOF/TOF (ultrafleXtreme, Bruker Daltonics) and MALDI-FT-ICR (solariX, 15T, Bruker Daltonics) MSI. An overview on the sample preparation is provided in S1 Fig.

### MSI data acquisition and initial processing

The MALDI-TOF was calibrated on internal metabolites [15], the FT-ICR was calibrated on DHB matrix clusters. Measurements were set up using the Bruker software FlexImaging 3.0 with a raster size of 100 μm. In MALDI-TOF MSI, the laser beam diameter was 50 μm, 300 laser shots were accumulated at each raster spot, laser power ranged from 44–47%. Signals were recorded from m/z 0–3000 in positive reflector mode with ion suppression up to m/z 50 and a resolution of 1 GS/s. At least two different seeds (biological replicates) of each sampling time point were analyzed by MALDI-TOF MS Imaging. In FT-ICR MSI, 200 laser shots (50 μm laser focus) were accumulated at each raster spot (100 μm) with 70% laser power, signals were recorded from m/z 200–2000. Co-registration between each MSI data set and its optical image was done with FlexImaging, which also served for inspection of single *m/z* intensity images (normalized to TIC). Based on the co-registered optical images, the regions of interest were outlined and semantic labels assigned (whole seed, endosperm, embryo, background). Average mass spectra were exported from FlexImaging or generated by ClinProTools. Peak picking was performed in FlexAnalysis (for single TOF MS) or in mMass for FT-ICR data [40].

### Compound identifications by FT-ICR data and MS/MS

Compounds were tentatively identified by accurate mass match of obtained high resolution FT-ICR m/z values to the *Metlin* database [41]. Identifications were confirmed using MALDI-TOF MS/MS on matrix coated barley tissue sections or on methanolic extracts. For extracts, 2 mg to 40 mg of freeze-dried, milled tissue were extracted with 1 ml water by ribolysing (3 x 45 s, 15 min pause, 6.5 m/s) using 500 mg ribobeads (0.5 mm diameter, Roth) for tissue disruption. Samples were centrifuged 15 min at 14,000 rpm. 1 μl to 2 μl of the supernatant were spotted on a ground steel target (Bruker Daltonics) with 1 μl 2,5-dihydroxybenzoic acid matrix (30 mg/ml in 50% methanol, 0.2% TFA). Dextrin 20 (Serva) with a concentration of 1 mg/ml was used as a reference sample for oligosaccharide analysis. The MALDI-TOF was calibrated on peptide standards. MS/MS was performed on selected precursor ions with individual laser settings using collision induced dissociation (CID) with argon as collision gas at a pressure of 2 x 10^6^ mbar and a collision energy of 8 keV. The software functions *Mass frontier* (Thermo scientific) and *IsotopePattern* (Bruker Daltonics) served for structure and formula elucidation of metabolites.

### Higher level analysis and visualization of spatio-temporal patterns in multiple MSI data sets

All of the following steps were implemented in python employing also established libraries for scientific computing and data mining [42–45].

Initially, all spectra are binned (sum of signals in window) to the same *m/z* axis to facilitate joint processing. The bin size of *Δ m/z* = 0.1 was chosen to be smaller than relevant *m/z* tolerances in later steps, but larger than the minimal distance between any of the *m/z* values in the raw data to avoid imputed intensities.

Next, all data sets are combined into a single data frame $M\in R^{m\times n}$, where each of the *m* rows contains all intensity values for a single spectrum *s* and each of the *n* columns represents the flattened intensity image *i* of a single *m/z* bin. A spectrum *s* is hierarchically indexed with the time point *t* ∈ *T* of its original data set, a compartment region label *r* and spatial position (*x*, *y*). Therefore, a single spectrum *s* can also be referred to as a signal pixel in a MS image of a sample from time point *t*. For the data set presented in this paper we used *T* = {1, 2, …, 8} and *r* = *{whole seed*, *embryo*, *endosperm*, *background}*. We will refer to the single MSI data set from time point *t* as *M*_*t*_ and with *M*_*t*,*r*_ to its sub-set labeled with *r*. $M_{t}^{mz}$ is the flattened intensity image for a single *m/z* bin and $M_{t,r}^{mz}$ accordingly the sub-image for region label *r*.

We will use the general term *molecular composition* to refer to the composition of molecules as represented by an analyzed spectrum *s* (or any aggregation of spectra, for example *M*_*t*_, *M*_*r*_, *M*_*r*,*t*_, *M*). After peak picking the set of all peaks in *s* may be referred to as a *peak profile*. If the molecules in question are metabolites, the term *metabolite (peak) profile* is used, otherwise the term (*molecular) peak profile* will be used.

The actual analysis is split into three steps: i. Preprocessing for each single data set *M*_*t*_, followed by joint preprocessing of all data sets *M*; ii. Joint clustering of *M*; iii. Interactive visual exploration and interpretation of results. Please see the S2 and S3 Figs for an overview.

### Preprocessing of MSI data sets

All data sets were square root transformed to account for heteroscedastic noise common in MALDI MSI [25]. Power and log transformations are useful alternatives but for our data sets square root transformation showed to be both effective and efficient. Subsequently all spectra were re-calibrated, filtered to a set of relevant spectra and m/z bins and then normalized to the median intensity of those peaks.

Since the calibration to internal metabolites was likely not perfect [25] an automated approach to correct small *m/z* shifts was employed. Such a step is commonly referred to as re-calibration in the context of MS. In the absence of ground truth, we employed the following qualitative heuristics: *m/z* shifts between two spectra *s*_*t*,*r*_ ∈ *M*_*t*,*r*_ and $s_{t,r´}\in M_{t’,r\prime }$ are less likely and smaller if they are from the same data set (*t* = *t*’) and molecular composition should be more similar if they share the same label (*r* = *r*′). The following pragmatic multi-pass approach was used to re-calibrate all spectra *s* ∈ *M*:

First, all spectra are re-calibrated to better match their region in their data set:, i.e. *s* ∈ *M*_*t*,*r*_ are shifted towards the median spectrum of *M*_*t*,*r*_. Then, all spectra are re-calibrated to their whole data data set signal distribution, i.e the spectra *s* ∈ *M*_*t*_ are shifted towards the median spectrum of *M*_*t*_. Last, all spectra are recalibrated according to the region-specific distribution, i.e. the spectra of all time points *s* ∈ *M*_*t*,*r*_ (*t* ∈ *T*) are shifted towards the all-time median spectrum of *M*_*t*,*r*_ (*t* ∈ *T*). The shifts in each pass are linear shifts of the *m/z* axis, which are computed using Fast Fourier Transform (FFT)-based cross-correlation between the intensities of the median-normalized spectra. Cross-correlation is influenced strongly by peak intensities, which may result in false corrections caused by matching unrelated high intensity peaks. The prior square root transformation together with the use of normalized spectra helps to avoid this problem. Furthermore, a fixed cut-off is used to limit shifts to small corrections with *Δ* m/z < 0.5 Da.

After re-calibration, intensity images and spectra considered not relevant to the analysis at hand are removed to reduce variability and the size of the data set. We refer to the spectra that are retained for analysis as *informative spectra* (based on the *informative peaks* introduced in Fonville [25] and adapt the preprocessing steps described in the same work as follows: All intensity images that positively correlate with known *m/z* values of the DHB matrix or the adhesive tape are removed. After that, spectra outside of the regions of interest are removed. For each of the remaining intensity images its randomness is assessed by computing the variance explained (VE) of the first component of the singular value decomposition (SVD) of the image rows. Images with a VE below the mean VE of all images are filtered out [25]. The remaining spectra and *m/z* bins are assumed to be informative regarding the biological variability in the regions of interest of the samples.

In the final preprocessing step all informative spectra are normalized to their median [25]. The resulting preprocessed data set is referred to as *P*.

### Unsupervised Clustering and MSI Segmentation

To analyze the complex interactions captured by comparative MSI some form of quantization of the *n*-dimensional feature space is required. Here, we consider each *m/z* bin as a dimension in this feature space and each spectrum (or signal pixel) is represented as a feature vector. While it is possible to use the complete preprocessed data set *P* for cluster analysis, we selected only a subset X* ⊆ *X* of the features by picking peaks based on *m/z* values of interest for this study. The resulting feature vectors ***x*** ∈ X* are referred to as peak profiles, since they represent only a targeted subset of the *m/z* bins of the informative spectra.

The Hierarchical Hyperbolic Self Organizing Map (H^2^SOM) was used for unsupervised clustering because it is fast and enables straightforward selection of a color code to generate meaningful cluster maps. It is based on Self Organizing Maps (SOM) but uses a regular lattice described in the hyperbolic and not in the Euclidean space and is trained hierarchically using beam search for faster computation [46]. During clustering the cosine distance was used to determine (dis-)similarities between feature vectors.

Since all peak profiles from all data sets (i.e. $x\in X_{t}^{*},$ (*t* ∈ *T*)) are clustered together and the number of peak profiles per data set $X_{t}^{*}$ can be different, the random sampling required for fitting the data was weighted to avoid sampling bias. The weights were chosen so each data set has the same probability of being sampled in any given step.

### Visualization for Interactive Exploration

The resulting segmentation of all data sets can be viewed and interactively explored using an updated version of our previously published Web-based hyperbolic Image Data Explorer (WHIDE) [46].

The central visualization is a segmentation map (also called cluster map) of the original data. This segmentation map is overlaid on the bright field images of the analyzed data sets. The color code of the segmentation map uses similar colors for areas with similar molecular peak profiles. Therefore, the user can easily see which areas share a similar composition. Such topology preserving color visualizations have also shown to be very useful in an early stage of high dimensional bioimage analysis for instance in the design of an appropriate pre-processing and signal normalization pipeline [47]. The whole process of visualization can be divided into the following three steps:

In the learning and color coding step (see S2 Fig), the algorithm computes a grid of nodes in the hyperbolic cluster space in which similar molecular peak profiles are represented by nodes close to each other and dissimilar molecular peak profiles are represented by nodes far from each other. This property can be achieved because the H^2^SOM, which is used to generate the segmentation, is trained to preserve the topology of the feature space in the lattice projection. Each node represents the typical molecular peak profile of one cluster in the data set.

This grid is projected from the hyperbolic space on a unit disk in Euclidean space with the Poincaré disk model (see [46] for details). The resulting disk-shaped grid approximates the properties of the original grid in hyperbolic space but can now be plotted in the user interface.

The hue-saturation-value (HSV) color system is used to assign colors to the nodes in the grid. To visualize this, a hue-saturation disk is plotted as a background of the disk. Thereby each node in the grid can be colored by the underlying color on the disk.

In the visualization step (see S3A Fig), the clustering of peak profiles is visualized as a cluster map. The cluster map is shown in the user interface as an overlay on top of the original bright-field images of the MSI data sets. The correspondence between clusters and pixels in the segmentation map is indicated by color: Each pixel in the cluster map is colored using the color of the corresponding grid node.

The resulting color code is a spectrum approximation, thereby offering more legible color steps–and thereby visibility for smaller details in the segmentation map–than simpler sequences like grayscale or two color sequences (e.g. red-blue) [48,49].

The combination of projection and color coding enables the interactive exploration (see S3B Fig) of the segmentation map. Simply rotating the color disk across the cluster nodes also rotates the color code of the segmentation map. This feature is very useful because the sensitivity for different color hues of human (and individual) vision varies. Only a low number of colors and thereby clusters can be used if all of them need to be recognized as distinct [49]. Furthermore, the user can change the focus on the grid with a fish-eye zoom to interactively navigate through the color space and to tune into a color mapping. This can be used to highlight clusters of interest and at the same time keeps smooth color transitions between similar clusters.

## Results

The results of our study are presented as follows. After summarizing observations during the preparation and experiments, we will present results of metabolite identifications by FT-ICR high resolution mass spectrometry and Time Of Flight MS/MS. After that, we will address spatio-temporal changes of oligosaccharides and antifungal metabolites. Third, we will finally present the data analysis results computed with the presented clustering algorithm and visually exploration using the WHIDE tool.

### Establishment of the seed sectioning procedure and sample preparation

During germination, the barley seeds exhibited typical developments with the softening of the endosperm after moistening and the growth of the embryo shoot and its roots (Fig 1). Using conventional cryosectioning and thaw mounting on the ITO slides, no intact sections could be obtained, since the endosperm did not adhere to the slide and disruptions of the entire section occurred frequently during sectioning. Adhesive film was used for the stabilization of the section [20]. The adhesive film allowed the reduction of slice thickness to 14 μm, fast transport for drying (>5 seconds), and fixation on the ITO slide (S1 Fig). However, kilned barley sections (malt) were of insufficient quality for MSI due to very high friability.

### MALDI MSI of barley seeds during germination

Metabolites were profiled by MALDI MSI in longitudinal and transversal orientations in mature barley and at seven time points during germination (Fig 1, S4 Fig). The number of detected compounds as well as signal intensities increased during germination from 50 m/z values (SN>5) at early germination (0d, S1d, G0d) up to 100 signals at later time points (G1d to G5d). A number of signals derived from the adhesive film and were excluded from further analyses. For the identification of metabolites, one representative sample (G2d) was analyzed using high resolution Fourier-Transform Ion Cyclotron Resonance (FT-ICR) MS imaging at Bruker Daltonics in Bremen, Germany. Database search (*Metlin* [41]) of the high resolution FT-ICR MSI data revealed potential compound annotations and MALDI-TOF MS/MS of tissue homogenates or of matrix-coated seed sections served for identification validation (S1A, S1B and S1C Table). In summary, 85 peaks were identified as [M+H]^+^, [M+Na]^+^, and [M+K]^+^ ions of 48 metabolites according to reporting standards for metabolomics [50] (Table 1). The identified metabolites were categorized in three classes, namely oligosaccharides, lipids, and secondary metabolites (Table 1). For detailed information about FT-ICR data and identifications see S1 Supporting Information (FT-ICR and TOF mass spectra), S1 Table (A: peak annotation and identification, B: FT-ICR peak list, C: *Metlin* search results), S2 Supporting Information (lipids), S3 Supporting Information (oligosaccharides) or Gorzolka *et al*. [15] (secondary metabolites).

**Table 1:** Detected and identified compounds from MS imaging of barley seeds.

**Lipids**	**Molecular formula**	**Molecular weight**	**[M+H]+**	**[M+Na]+**	**[M+K]+**	**Identification level**
choline	C5H13NO	103.11	**104.11**	126.09 ^n.d.^	142.06 ^n.d.^	3
phosphocholine	C5H14NO4P	183.07	**184.07**	206.06 ^n.d.^	222.03 ^n.d.^	3
PC(16:0)	C24H50NO7P	495.33	**496.34**	**518.32**	**534.09**	2
PC(18:2)	C26H50NO7P	519.33	**520.34**	542.32 ^n.d.^	558.30 ^n.d.^	2
PC(32:0)	C40H80NO8P	733.56	**734.57** ^n.d.^	**756.55** *	772.53 ^n.d.^	3
PC(34:3)	C42H78NO8P	755.55	**756.55**	778.53 ^n.d.^	794.51 ^n.d.^	3
PC(34:2)	C42H80NO8P	757.56	**758.57**	**780.55** *	**796.52**	2
PC(34:1)	C42H82NO8P	759.58	760.59 ^n.d.^	**782.57**	798.54 ^n.d.^	2
PC(36:5)	C44H78NO8P	779.55	**780.55**	802.54 ^n.d.^	818.30 ^n.d.^	3
PC(36:4)	C44H80NO8P	781.56	**782.66** *	804.55 ^n.d.^	**820.53**	2
TG(52:2)	C55H102O6	858.77	859.77 ^n.d.^	881.76 ^n.d.^	**897.73**	3
PI(40:3)	C49H89O13P	916.60	917.62 ^n.d.^	939.60 ^n.d.^	**955.57**	3 (> 1ppm)
PI(42:5)	C51H89O13P	940.60	941.62 ^n.d.^	**963.60**	**979.57**	3 (> 1ppm)
**Carbohydrates**	**Molecular formula**	**Molecular weight**	**[M+H]+**	**[M+Na]+**	**[M+K]+**	
2 hexoses	C12H22O11	342.12	343.12 ^n.d.^	365.11 ^n.d.^	**381.08**	2
3 hexoses	C18H32O16	504.17	505.17 ^n.d.^	**527.16**	**543.13**	1
4 hexoses	C24H42O21	666.22	667.23 ^n.d.^	**689.21**	**705.18**	1
5 hexoses	C30H52O26	828.27	829.28 ^n.d.^	**851.26**	**867.24**	1
6 hexoses	C36H62O31	990.33	991.33 ^n.d.^	**1013.31**	**1029.29**	1
7 hexoses	C42H72O36	1152.38	1153.39 ^n.d.^	**1175.37**	**1191.34**	1
8 hexoses	C48H82O41	1314.43	1315.44 ^n.d.^	**1337.42**	**1353.40**	1
9 hexoses	C54H92O46	1476.49	1477.49 ^n.d.^	**1499.47**	**1515.45**	1
10 hexoses	C60H102O51	1638.54	1661.53 ^n.d.^	**1661.53**	**1677.51**	1
11 hexoses	C66H112O56	1800.59	1801.60 ^n.d.^	**1823.58**	**1839.56**	1
12 hexoses	C72H122O71	1962.64	1964.12 ^n.d.^	**1986.10**	**2001.74**	1
13 hexoses	C78H132O76	2124.70	2126.07 ^n.d.^	**2148.05**	**2163.91**	1
**Hydroxycinnamic acid amides / hordatine precursors**	**Molecular formula**	**Molecular weight**	**[M+H]+**	**[M+Na]+**	**[M+K]+**	
*p-*coumaroylagmatine	C14H20N4O2	276.16	**277.17**	299.15 ^n.d.^	315.12 ^n.d.^	2
coumaroyl-hydroxyagmatine	C14H20N4O3	292.15	**293.16**	315.14 ^n.d.^	331.12 ^n.d.^	2
feruloylagmatine	C15H22N4O3	306.17	**307.18**	329.16 ^n.d.^	345.13 ^n.d.^	2
feruloyl-hydroxyagmatine	C15H22N4O4	322.16	**323.17**	345.15 ^n.d.^	361.13 ^n.d.^	2
**Hordatines**	**Molecular formula**	**Molecular weight**	**[M+H]+**	**[M+Na]+**	**[M+K]+**	
hordatine A	C28H38N8O4	550.30	**551.31**	**573.29**	**589.26**	2
hydroxy-hordatine A	C28H38N8O5	566.30	**567.30**	**589.29** *	**605.26**	2
hordatine B	C29H40N8O5	580.31	**581.32**	**603.30**	**619.28**	2
hydroxy-hordatine B	C29H40N8O6	596.31	**597.31**	**619.30** *	**635.27**	2
hordatine C	C30H42N8O6	610.32	**611.33**	633.31 ^n.d.^	649.29 ^n.d.^	2
hydroxy-hordatine C	C30H42N8O7	626.32	**627.32**	649.31 ^n.d.^	665.28 ^n.d.^	2
hordatine A + Hex	C34H48N8O9	712.35	**713.36**	**735.34**	**751.32**	2
hydroxy-hordatine A + Hex	C34H48N8O10	728.35	**729.36**	**751.34** *	**767.31**	2
hordatine B + Hex	C35H50N8O10	742.36	**743.37**	**765.35**	**781.33**	2
hydroxy-hordatine B + Hex	C35H50N8O11	758.36	**759.37**	**781.35** *	**797.32**	2
hordatine C + Hex	C36H52N8O11	772.38	**773.38**	**795.36**	**811.34**	2
hydroxy-hordatine C + Hex	C36H52N8O12	788.37	**789.38**	**811.36** *	**827.33**	2
hordatine A + 2 Hex	C40H58N8O14	874.41	**875.41**	**897.40**	**913.37**	2
hydroxy-hordatine A + 2 Hex	C40H58N8O15	890.40	**891.41**	**913.39** *	**929.37**	2
hordatine B + 2 Hex	C41H60N8O15	904.42	**905.43**	**927.41**	**943.38**	2
hydroxy-hordatine A + 2 Hex	C41H60N8O16	920.41	**921.42**	**943.40** *	**959.38**	2
hordatine C + 2 Hex	C42H62N8O16	934.43	**935.44**	**957.42**	**973.39**	2
hydroxy-hordatine A + 2 Hex	C42H62N8O17	950.42	**951.43**	**973.41** *	**989.39**	2
**Unknowns**	**Molecular formula**	**Molecular weight**	**[M+H]+**	**[M+Na]+**	**[M+K]+**	
*m/z* **74.65**	-	-	-	-	-	4
*m/z* **118.28**	-	-	-	-	-	4
*m/z* **175.33**	-	-	-	-	-	4
*m/z* **230.00**	-	-	-	-	-	4
*m/z* **558.18**	-	-	-	-	-	4
*m/z* **650.55**	-	-	-	-	-	4
*m/z* **674.38**	-	-	-	-	-	4
*m/z* **1006.58**	-	-	-	-	-	4
*m/z* **2200.69**	-	-	-	-	-	4

Bold m/z values: detected and used for cluster analyses, n.d.: not detected,

*: refused in cluster analyses due to nearly isobaric ions.

Lipids: PC: phosphatidylcholine; PE: phosphatidylethanolamine; PE-NMe: N-methyl-phosphatidylethanolamine; PI: phosphatidylinositol; TG: triglyceride. Brackets: total number of C-atoms in the fatty acids with the number of double bonds.

Carbohydrates: degree of polymerization with the number of hexose residues.

Identification level: affirmation of identification in accordance to the minimum reporting standards [50]. 1) Identified [MS/MS of compound and standard compound], 2) Annotated [MS/MS of compound, reference to literature], 3) Putative [no MS/MS available, database hit of high resolution FT-ICR data], 4) Unknown.

In the next section, the observations for oligosaccharides and antifungal secondary metabolites will be summarized. Detailed information on the localization of lipids is given in S2 Supporting Information. Briefly, lipids were detected in the endosperm with a distinction between monoacyl phosphatidylcholines, which appeared in the endosperm, and diacyl phosphatidylcholines, which demonstrated dynamic localization changes during germination at the aleurone layer and in the endosperm.

### Localization of oligosaccharides

**Fig 2:**
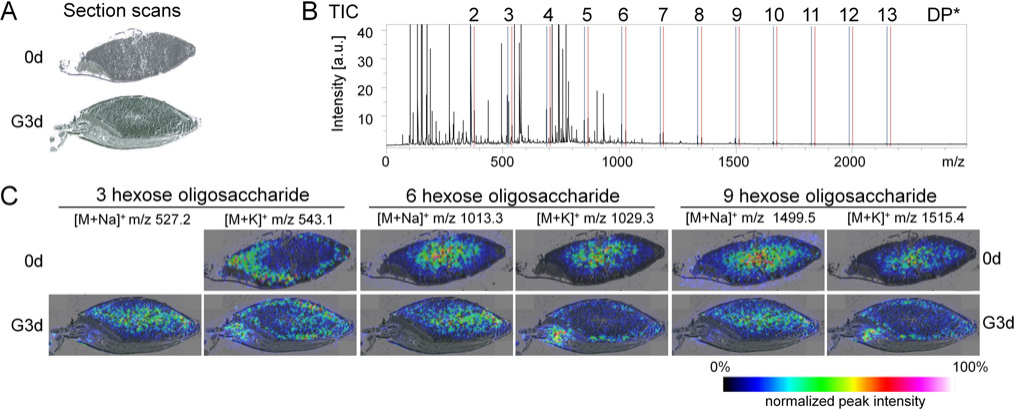
Localization of oligosaccharides in barley during germination. A) Cryo-sections of non-germinated (0d) and three day germinated (G3d) barley. B) Average mass spectrum of all MS acquired from germinated barley with [M+Na]^+^ (red) and [M+K]^+^ (blue) ions of oligosaccharides. *DP: degree of polymerization. C) Intensity heat maps of oligosaccharides with three, six, and nine hexoses in sodium [M+Na]^+^ and potassium [M+H]^+^ adducts in ungerminated barley (0d) and after three days of germination (G3d). MS intensities were normalized to the TIC of each mass spectrum; the highest relative intensity of all MS was set to 100%. The distributions of these compounds at all time points of the germination process are provided as S5 Fig.

Oligosaccharides with two to thirteen hexose units (degree of polymerization DP = 2–13) were detected as sodium and potassium adducts. Fig 2 depicts the localizations of three oligosaccharides in non-germinated and germinated barley, which is representative for early and late germination time points. A table of all time points is provided as S5 Fig. At early germination, sodium and potassium adducts co-localized in the central endosperm. At later germination time points, the potassium adducts were detected near the aleurone layer, the sodium adducts exhibited highest intensities in the central endosperm regions. These distinct localizations of adducts were observed and superimposed for all degrees of polymerization. The merge of both adducts underlined their complementary localizations (S6 Fig). After the first day of germination, oligosaccharides were detected in the embryo with a localization in the embryo center close to the scutellum in elongated shoots (S5 Fig).

### Localization of antifungal secondary metabolites: hydroxycinnamic acid amides and hordatines

**Fig 3:**
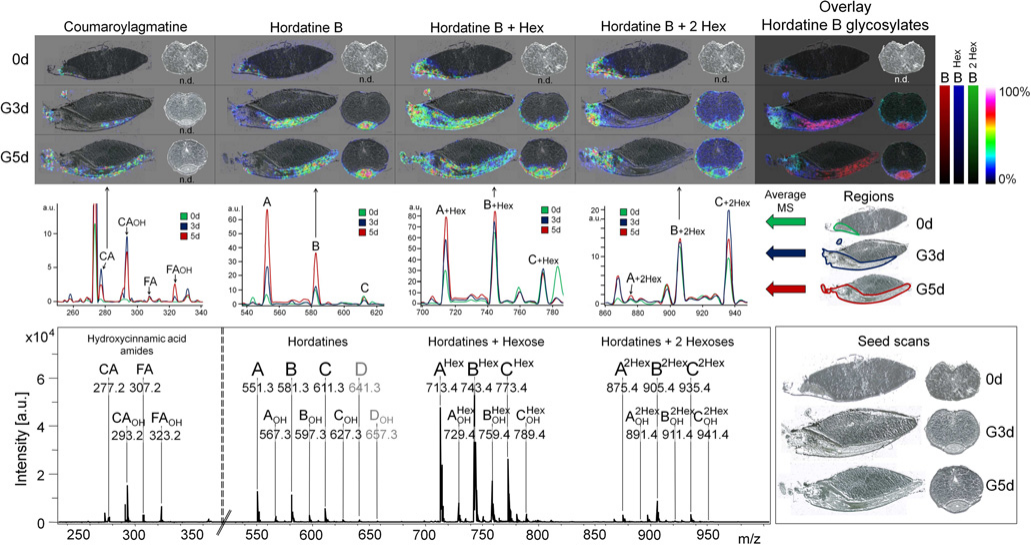
Localization and signal intensities of hydroxycinnamic acid derivatives and hordatines in germinating barley. Top: Localization of p-coumaroylagmatine (CA) as representative for hydroxycinnamic acid amides and hordatine B as representative for hordatines that co-localized to hordatine B when occurring in the same modification state. Intensity maps depict the non-glycosylated (*m/z* 581), glycosylated (*m/z* 743), and disaccharide-modified form (*m/z* 905) at three time points during germination (0d: non-germinated barley, G3d: three days germinated, G5d: five days germination) in longitudinal and transversal section plane. Hordatines were not detected in cross sections in non-germinated barley. The last panel shows an overlay of the three modification forms. Ion intensities were normalized to the TIC, the highest relative intensity was set to 100%. Middle panel: Average mass spectra from annotated embryo measurement regions (right) in non-germinated (green), three days (blue) and five days (red) germinated barley. Bottom: Mass spectrum with indicated peaks of hydroxycinnamic acid amides as hordatine precursors (*m/z* 250–350) and of hordatine A, B, C, and D (D not detected, grey font), hydroxylated hordatines (-OH), and hexose-modified derivates (Hex / 2 Hex) at *m/z* 550–1000. CA: coumaroylagmatine, FA: feruloylagmatine, CA-OH / FA-OH: hydroxylated CA and FA.

In a previous study, 20 hordatines and hydroxycinnamic acid amides (HCAA) were identified in barley [15]. Here, the time dependent localizations and signal intensity changes of hordatines and HCAAs during germination are investigated (Fig 3). HCAAs, which are antifungal compounds as well as precursors for hordatines, were mainly detected in the roots of the seeds except of coumaroylagmatine (CA), which also occurred in the barley shoot (Fig 3). During germination, the signal intensities increased in longitudinal sections, whereas in transversal sections, none of the HCAAs was detectable. In general, the signal intensities of the monomeric HCAAs were low in comparison to their dimers (hordatines).

The hordatines of the same glycosylation state demonstrated co-localization patterns (S7 Fig). This is shown in Fig 3 for the hordatine B in its non-glycosylated, glycosylated, and maltosylated form. At early germination (0d, G0d, and G1d), hordatine signals were of very low intensity and localized in the embryo with minor distinctions between the modified forms. With the differentiation of embryo substructures, the hordatines segregated in their localization. Non-glycosylated hordatines were detected in the inner shoot, with an accumulation at the upper shoot regions at late germination (G5d). Glycosylated hordatines were present in the outer shoot tissues as well as in the roots. This inverse distribution was even more apparent in transversal sections and in the overlays (Fig 3). The disaccharide-modified compounds were mainly detected in the roots without any significant changes during germination. These compounds were low in the transversal sections, only the maltosylated hordatine B was detected, which co-localized with the shoot surrounding glycosylated derivatives. The transversal sections indicated the presence of glycosylated hordatines under the husk, which was also observed for non-glycosylated compounds at early germination (see S7 Fig).

During germination, the hordatine signal intensities increased constantly (Fig 3) and after two days of germination, they were among the highest peaks of the overall mass spectrum. Interestingly, the intensity proportions between the hordatine peaks changed with the glycosylation: Non-glycosylated compounds exhibit highest hordatine A signals, followed by B and C. With one hexose residue attached, hordatine B was the highest peak, followed by A and C. If maltosylated, hordatine C is of highest abundance, followed by B and A.

The inspection of oligosaccharides, lipids and secondary metabolites revealed their highly tissue-specific localization. Average mass spectra of the main compartments embryo, root, scutellum, and endosperm underlined the different metabolic compositions and pointed to unique compounds for each compartment (S8 Fig). High hordatine signals were characteristic for the embryo tissues with differential proportions of hordatine A, B, and C in the shoot and root (S8 Fig) and coumaroylagmatine and feruloylagmatine as root specific signals. The endosperm subdivision revealed distinct distributions of phosphatidylcholines with higher abundances of monoacyl PCs in the apical parts (S8 Fig, endosperm 2) and diacyl PCs near the scutellum (S8 Fig, endosperm 1).

### MSI cluster analysis for the description of metabolite profiles during germination

All mass spectra were preprocessed with the same methods given in materials and methods to achieve comparability. The initial binning and the preprocessing reduced the size from ~83,000 *m/z* values and ~28,000 spectra in the raw data to ~5,000 *m/z* bins and ~20,000 spectra in *X*. Based on the in-depth analysis of single data sets and peak identifications, 101 *m/z* values (see Table 1, black font) of interest were picked from *X*. Several bins were too close together to be separated by the automatic preprocessing and only one of each peak pair (lower *m/z* value) was retained to avoid redundant features (as indicated with asterisks in Table 1). The resulting data set X* (~20,000 peak profiles with intensities for 93 *m/z* peaks) was clustered and visualized to detect co-localizing metabolites and to relate emerging profiles to their corresponding spatial segments on the tissue.

**Fig 4:**
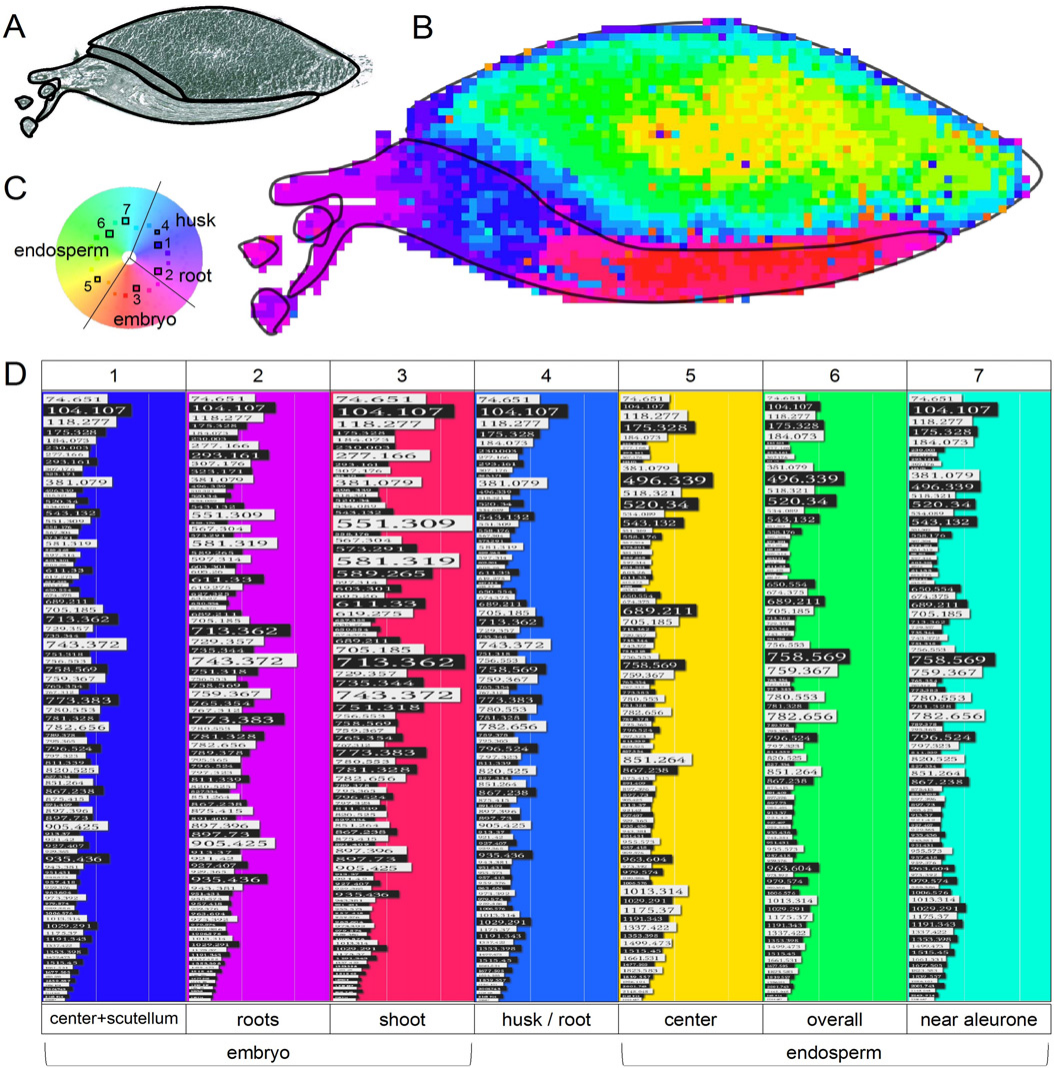
Unsupervised spatial segmentation of 93 *m/z* intensities obtained from MALDI MSI of a three days germinated barley seed (G3d). A: Image scan with outlines for the labeled seed compartments. B: Cluster map with the main compartments of the seed, transferred from A. C: Cluster distance wheel with 32 clusters set for this analysis; the 7 peak profile clusters presented in D are indicated with boxes. D: Mass lists of representative clusters; the bar size is indicative for the signal intensities; *m/z* identifications are provided in Table 1. See https://ani.cebitec.uni-bielefeld.de/barleymsi for an interactive version of the results in the WHIDE tool.

Fig 4 shows the result from the cluster analysis of a three days germinated barley seed. The metabolic profiles were represented by distinct clusters, which show strong correlation to the seed compartments. In comparison to tissue profiling with average spectra from regions of interests (see S8 Fig), this clustering revealed metabolic heterogeneity with more detail within the tissues at single positions. The embryo area was classified in shoot inner layers (Fig 4, red), shoot outer layers (Fig 4, pink), embryo center (Fig 4, blue) and two different root peak profiles (violet) (Fig 4, peak profile clusters 1–4, for m/z identities refer to Table 1), which was mainly based on hordatine and lipid signals. The embryo center, metabolite patterns were similar to the scutellum with a mixture of hordatine and oligosaccharide signals. The endosperm MS patterns were differentiated by the proportional abundances of oligosaccharides and mono- and diacyl phosphatidylcholines (yellow, green; profiles 5–7). These endosperm clusters were arranged in a circuit gradient from the center towards the aleurone layer. The aleurone layer itself was represented by a one or two MS thin line and exhibited an embryo-center like metabolite composition (Fig 4, blue).

**Fig 5:**
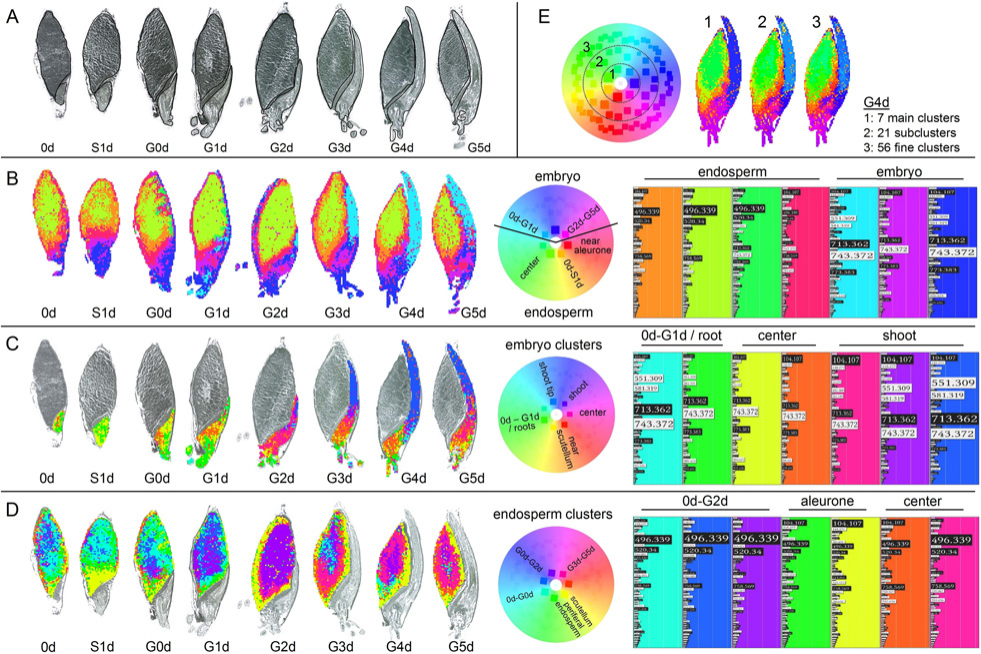
Unsupervised spatial segmentation of eight independent MALDI MSI analyses covering the first days of barley germination using WHIDE. A: Image scans with outlines of labeled seed compartments. B: Cluster analysis of all mass spectra of the whole grain areas. C: Cluster analysis of all mass spectra of the embryo areas as annotated in A. D: Cluster analysis of all mass spectra of the endosperm as annotated in A. E: Effect of the cluster granularity (7, 21, 56 clusters) on mapping results, exemplarily shown for four days germinated barley (G4d) from analysis B. The visualized cluster, granularity was set to 7 in A, B, and C to assign clear cluster profiles (right panels). 93 *m/z* values were selected for spatial segmentation in all analyses. Their contribution to the distinct clusters is indicated by the bar size in the right panels; for *m/z* identification see Table 1. For an interactive exploration of the results in WHIDE see https://ani.cebitec.uni-bielefeld.de/barleymsi.

Next, to resolve temporal developments and to map the metabolite profiles on the seed sections, all time points of the germination process were analyzed in one batch with one representative replicate each and a comprehensive overview on the results is given in Fig 5, starting with a morphological overview on the samples in Fig 5A. The WHIDE visualization revealed again a distinction of the embryo and the endosperm peak profiles (Fig 5B). Time-dependent changes could be observed in the endosperm with an early germination metabolite profile (orange in Fig 5B) with high abundances of mono- and diacyl PCs (*m/z* 496, 520, 758), which changed to a later germination pattern (green in Fig 5B) with the same main masses but distinct proportional abundances. Higher contents of the choline fragment (*m/z* 104) and of oligosaccharides caused the generation of additional endosperm profiles (red–pink in Fig 5B) near the scutellum and the aleurone layer. The embryo clusters (blue and pink in Fig 5B) revealed a tissue-specific split after the differentiation of embryo morphology with light blue at the shoot tip, dark blue at the lower parts and the roots and pink at the embryo center. The most prominent *m/z* were glycosylated hordatines (*m/z* 713, 743) with cluster differentiations by the presence of oligosaccharides at the embryo-scutellum interface (Fig 5B, pink).

One special feature of the H^2^SOM algorithm is the hierarchical structure in the cluster result, so the clusters (i.e. peak profile prototypes) are organized in four levels with an increasing number of clusters: 1 (root node), 7 (first layer), 21 (second layer) and 56 (third layer). Each parent cluster is connected to subclusters in the subsequent level. This allows a visualization of the patterns on different levels of cluster granularity which relates to resolving higher (i.e. visualizing the colors of all clusters) or lower levels (i.e. visualizing the colors only of the seven parent first layer clusters) of detail in the peak profiles (Fig 5E). The visualization of the finer subclusters in the third layer revealed some substructures within the embryo, but mostly introduced no further information that was not already present in the second layer. Altogether, the seven clusters in the first layer already represented the main features and are therefore referred to as *main (peak profile) clusters*. In Fig 4, we restricted the coloring to these main clusters to increase the color contrast in the segmentation map and thereby enhance the perception of spatial patterns for the most expressed peak profiles.

The variations in the globally expressed metabolite profiles are small in comparison to the variance between the embryo and endosperm regions. Two separate cluster analyses were performed for all embryo peak profiles (Fig 5C) and all endosperm peak profiles (Fig 5D) to focus on the variability within each compartment. This resulted in a much more detailed view on the tissue specific metabolic profiles and their localizations, since the clusters of these compartments are now shown with more color contrast, making the inner compartment variation visually accessible.

In the embryos, only two of the seven main clusters were present at early germination stages (0d–G0d). These profiles correspond to patterns observed in developed roots. The discriminating features are proportions of hordatines (*m/z* 551, 581, 713, 743, 773, 905, 935), coumaroylagmatine (*m/z* 277) and feruloylagmatine (*m/z* 293) and the low abundance of the choline head group (*m/z* 104). After root penetration through the hull (G0d, G1d), further clusters (orange, yellow in Fig 5C) arose and a metabolic segregation between roots, center, and shoot became apparent. After G2d, the growing embryo provided a constant differentiation into the seven main clusters, which were in agreement with morphological and developmental stages. The shoot tip contained higher levels of non-glycosylated hordatines (mainly hordatine A and B, blue in Fig 5C), the mid shoot part segregated by lower hordatine A and B levels, but higher choline fragment contents (*m/z* 104, purple in Fig 5C). Near the scutellum (orange in Fig 5C), the hordatine proportions changed towards glycosylated derivates with higher contents of hordatine B (*m/z* 743) and C (*m/z* 773) than in the upper shoot, which were also the main features for the roots (green in Fig 5C) segregation.

In the endosperm, a constant pattern segregation was observed during the whole germination process, which was the distinction between the central endosperm with high contents of monoacyl phosphatidylcholines (*m/z* 496, *m/z* 520) and endosperm areas near the scutellum and the aleurone layer (green and yellow), which exhibited higher proportional abundances of the choline head group (*m/z* 104) and a distinct pattern of oligosaccharides. In the endosperm center, a time dependent cluster development could be observed with early germination patterns (0d–G1d, blue in Fig 5D) and late germination patterns (G2d–G5d, purple and pink in Fig 5D). This switch was mainly due to the increasing lipid proportion at later germination states, which could even be subdivided in concentric circles around the inner endosperm (G3d–G5d, blue / pink / orange / yellow; Fig 5D).

## Discussion

Within this study, the cereal germination process was analyzed by MALDI MSI over time. A novel, easy and inexpensive method was developed, that allowed sectioning, mounting, and processing of the hard and friable cereal seeds. The protocol is certainly applicable to numerous other hard or crumbly tissues; therefore, it expands the range of potential analytical targets for MSI. DHB was a suitable matrix for metabolite profiling of barley phospholipids, carbohydrates [9], and a broad range of secondary metabolites. Following our sectioning protocol and quality measures, localizations as well as proportional signal intensities were highly reproducible.

More than 80 signals could be identified by high-resolution FT-mass spectrometry in combination with MALDI-TOF MS/MS. This is a comprehensive list, especially in MSI of plants, where most previously published studies focused on specific metabolite classes [9,12,13,19]. However, in comparison to chromatography-based analyses, this list appears restricted: While GC-MS [8] and LC-MS based metabolomics provide information on several hundreds of metabolites at a glance, MS Imaging approaches suffer from sensitivity and distinction capacities between isobaric compounds (e.g. many lipids). In addition, the on-tissue extraction of ions is highly influence by the properties of the tissue itself: Ion abundances influence the adduct formation (see sodium and potassium adduct localization of oligosaccharides), ion suppression due to high abundant co-localized metabolites or metabolites might be bound to tissue structures. These tissue and ionization effects might also explain the astonishing observation, that lipids were mainly detected in the endosperm of barley but not in the embryo, although the embryo exhibits even higher proportional lipid contents (embryo ~20% lipids, bran-endosperm: ~ 3–7% lipid content [51,52]). A) Mainly charged phospholipids were detected, but nearly no unpolar lipids due to their poor ionization; therefore, a broad part of the lipidome is not covered in this analysis. B) The lipids in the embryo and aleurone layer are organized in oilbodies that are accompanied by oleasome proteins [53], whereas in the endosperm, they are either starch associated or non-starch lipids [54]. These associated biological matrices might influence the lipid ionization and detection. C) The embryo exhibits high contents of charged secondary metabolites (hordatines), which might cause ion suppression of lipids. The use of different MS detectors, ionization methods and sample preparation methods (e.g. in MALDI-MS the matrix and solvent) would certainly complement each other and broaden the spectrum of detected compounds (see [11–13] for more details) to enable a more comprehensive view on the metabolism.

MS/MS was not suitable for the absolute identification of all chemical structures. For oligosaccharides, starch (α-1,4 or α-1,6 linkage) and cellulose (β-1,4 linkage) are both targets of endosperm degradation [5]. The MS/MS analysis of the resulting oligomers would reveal hexose fragments in all cases, therefore, those compounds could not be distinguished using MALDI MS. However, all hexose oligomers are indicators for ongoing endosperm hydrolysis. For lipids, mainly phosphatidylcholines were detected and could be annotated by the occurrence of the phospho-choline head group in the MS/MS. The exact fatty acid composition could not be assigned due to a) the MALDI-TOF MS/MS sensitivity and b) the co-occurrence of isobaric lipids of different fatty acid structures. Linoleic acid is the most abundant fatty acid in the barley seed [5] and it is highly likely that most identified PCs contained this double-unsaturated fatty acid. It is astonishing that the number of lipids found in the embryo is relatively low. The localization of lipids could be biased by the poor ionization of triglycerides. The higher number of lipids localized to the endosperm could be an effect of the metabolic turnover of triglycerides that are transported to the embryo. For hordatines, UHPLC-MS/MS provided very accurate and reliable identifications [15], which were independently validated by another study [55].

Oligosaccharides were detected with different localizations of sodium and potassium adducts, but with uniform distributions after merging the two mass values. This emphasized the need for the annotation of paired peaks that derived from the same compound, which severely challenges quantification. In other MALDI MSI approaches, differential adduct formation was addressed by adding sodium or potassium salts to the matrix solution to force ion formation of only one adduct peak [54] or by washing the tissue with hydrophilic solvents [56,57]. Tissue washing was refused in this study, as hydrophilic solvents would have caused metabolite diffusion and high amylase activity [58]. MS signal intensities were not suitable for absolute quantification, which is still a challenge in MALDI MSI [59]. The use of labeled external standards might be beneficial to address the quantification of oligosaccharides.

Despite the need for further optimization for quantitative conclusions, the presented data demonstrate that MALDI MSI is suitable to monitor ongoing endosperm degradation. The hydrolysis of endosperm cell walls, starch, and storage proteins is essential to provide energy and building blocks for the growth of the embryo. This hydrolysis starts in proximity to the embryo and progresses during germination to the center and the distal parts of the endosperm [5]. Interestingly, this degradation pattern was reflected by the potassium adducts of oligosaccharides and even more pronounced in cluster analyses with circular cluster arrangements in the endosperm, which indicated the gradual change of metabolite patterns from the aleurone layer towards the inner endosperm. Increased potassium contents seemed to accompany increased amylolytic, proteolytic or otherwise hydrolytic enzyme activity. In the embryo, oligosaccharides increased near the scutellum during germination. The translocated saccharides from the endosperm might have been polymerized again in the embryo to build cell walls, as proposed for germinated rice embryos [60].

Mono- and diacyl phosphatidylcholines exhibited distinct localizations. Monoacyl PCs were detected in the endosperm throughout the malting process without significant changes. In contrast, diacyl PCs were detected in the scutellum and in the husk or aleurone layer in ungerminated barley, which corresponded to observations in ungerminated rice seeds [20]. During germination, diacyl PCs occurred in the endosperm near the scutellum and the aleurone layer, which covered the distribution of the oligosaccharide potassium adducts; thus, also the diacyl PCs displayed the progression of endosperm degradation. The diacyl PCs might have already been present in the endosperm cell membranes at early germination time points, but undetectable or not extractable. The hydrolysis of the starch, structural proteins, and the cell walls during germination may have uncovered the cell membranes and therefore enhanced the extraction, ionization, and detection of the diacyl PCs, whereas the detection of monoacyl PCs was not significantly affected.

Hordatines provided very tissue-specific localizations, which corresponded to their glycosylation. Hordatine A and B derive from the dimerization of coumaroylagmatine and feruloylagmatine [61], exhibit antifungal properties [62] and can be glycosylated [63]. The other hordatine derivatives are dimers of hydroxycinnamic acids in diverse combinations [15] and might exhibit similar biological functionalities. Regarding their potential role in pathogen defense, their localizations and glycosylations are of high importance for agriculture. During germination, the overall hordatine signals increased, which was consistent with quantitative analyses [64]. The functionality of hordatine A, B, C, and D might be various, which is implied by the different distributions between roots and the shoot: Hordatine A is higher in the shoot, hordatine B higher in the roots, which might reflect specific intrinsic (e.g. developmental) or extrinsic (e.g. stress-induced) reactions of the two organs. In addition, the intensity patterns of the glycosylated hordatines did not reflect the intensity patterns of the non-glycosylated hordatines as visible in the metabolite clusters analyses. Therefore, glycosylation appears to be a controlled, tissue specific process. Roots face a different environment during germination with direct contact to pathogens, herbivores and abiotic conditions such as drought stress, whereas the shoot is protected by the hull. The differential functionality of the hordatines A, B, C and D might be additionally changed or fine-tuned by glycosylation to meet those differences. The glycosylated hordatines split in their localization after the first and second day of germination in consistency with the macroscopic development of the embryo organs. Here, maltosylated hordatines (especially the B form) might be essential in the root for optimal growth and pathogen protection. In the shoot, the outer (older) leaves exhibited more glycosylated compounds than the younger inner leaves, so the expression and activity of glycosyltransferases might depend on the age of the tissue or might be induced by the tension and friction of the leaves during growth under the husk. Therefore, glycosylation might be either environmentally controlled as well as a genetically fixed pattern. More functional information is needed for a biological discussion of these observations, but the very specific localizations and tissue-specific hordatine compositions highly imply differences in their biological roles and functionalities.

In contrast to the barley-specific hordatines, hydroxycinnamic acid amides are widespread in plants and functionally characterized (e.g. in Arabidopsis [65] and potato [66]). Amongst many other functions (mainly in plant development), HCAAs play an important role in pathogen defense [67,68]. In barley, they increased upon jasmonate and abscisic acid (ABA) treatment [69] and coumaroyl-hydroxyagmatine accumulated in barley leaves upon infection with *Erysiphe graminis f*. *sp*. *hordei* [70]. Three of the four detected HCAAs were mainly localized in the roots, which suggested their specific functionality in the root tissues. As discussed for hordatines, the HCAAs might provide specified protection of the young rootlets against infection, might also fortify and stabilize the cell walls for soil penetration and might support drought resistance. Although HCAAs are the precursors of hordatines, their localization was distinct. Considering their wide range of functionalities [71], their distributions are determined by many other factors despite hordatine biosynthesis.

### Analysis of the barley germination process using MSI cluster analysis

This study presented comparative cluster analysis of several independent MSI experiments. The specialized preprocessing improved spectral alignment and normalization and allowed to assign metabolite profiles to small tissue structures such as the aleurone layer (Fig 5), which was advantageous over tissue profiling (S8 Fig), where small structures might be lost due to spectra averaging. Using the WHIDE web tool, time-dependent metabolic developments over all sampled stages of germination could be simultaneously visualized, for the time enabling a comparative visual exploration. This revealed the molecular heterogeneity of macroscopically uniform tissue like the endosperm. Furthermore, the clusters displayed biologically highly relevant features such as the endosperm hydrolysis and embryo axis subdivision. On-tissue cluster mapping reduced the multidimensional dataset and allowed to extract co-localizing features like hordatines and oligosaccharides. WHIDE provides an easy read-out of the characteristic *m/z* values (as illustrated in Fig 4D) and intuitive evaluation of similarities (as illustrated in Fig 4C).

The embryo and the endosperm fulfill very different physiological functions during germination, which was reflected by the large metabolic distance between their respective cluster groups. Only small changes within these two feature groups could be resolved, when the whole area of seeds was analyzed. Consequently, single seed organs were clustered separately to zoom into time-dependent changes. This provided a detailed annotation of time- and tissue dependent metabolite clusters of the growing embryo.

In the last germination states (G2d–G5d), highly reproducible tissue-specific subclusters were generated, mainly based on the proportions of hordatines in their non-glycosylated, glycosylated, and maltosylated forms, hordatine A, B, and C intensities, as well as the occurrence of oligosaccharides and lipid fragments. The functions of these compounds and their single localizations were already discussed before. Here, the special metabolite combination (patterns) provided tissue signatures, which were not represented by single *m/z* localizations, e.g. the subdivision of the embryo in seven, 21 or even 56 metabolic clusters. It should be emphasized, that the embryo subclusters changed gradually along its shoot axis and that these clusters appeared in the same order in the distance wheel, which indicated a constant metabolic fine-tuning of the embryo tissues in hordatines, hordatine modifications, oligosaccharides, and lipid contents and proportions. The advantage of metabolic signatures over single *m/z* localizations for additional tissue subdivision was also apparent in the endosperm. Single *m/z* values correlated with reported hydrolase activity during germination [5] and therefore indicated the progression of endosperm breakdown. However, the concentric rings of several clusters stressed this biological process, revealed a much finer spatial resolution of the metabolic states within the tissue and provided information about the proportions of lipids and oligosaccharides that were indicative for each state.

Choosing the H^2^SOM algorithm for spatial segmentation enabled a straightforward visualization of all clusters on an interactive cluster map, because the clusters and their similarities can be represented by a graphical model that enables both a meaningful color code for initial overview and relative modifications of that color code in a tight-feedback loop to explore the segmentation in more detail. The color coding is considered meaningful, since a circular and continuous color scale is applied. The circularity feature is important to reflect the fact, that the displayed variable, the cluster, is not an ordered attribute (i.e. there is no lesser / greater relation defined) and there is no upper and lower end of the attribute value. Second, the continuity of the color scale is important to reflect the fact the cluster prototypes describe a data distribution rather than discrete states, so we do not consider it as a qualitative attribute, which would be better visualized using other color mapping techniques.

To select appropriate features for the cluster analysis best-practice preprocessing steps were adapted to the case of comparative MSI analysis. While the evaluation of the preprocessing and the biological interpretation of the cluster result support our assumptions that sufficient spectral correspondence can be established for this data set, the spectral resolution and some lack of even more identifications leaves a minor degree of uncertainty. It is central to evaluate any preprocessing and analysis strategy on a per data set basis until established methods to measure MSI data quality exist [71].

While automated peak picking might have resulted in even more signals for analysis, the existing list of relevant and at least partially identified metabolites was chosen to make the already complex cluster result easier to interpret. This list merged the peaks from all time points and excluded background artifacts from analyses. In addition, the manual peak inspection prevented overrepresentation of features by failing isotope annotation, because isotope picking is critical to automate in lower mass resolution mass spectrometry like TOF and especially in MS imaging,

To our knowledge, this is the first attempt to analyze multiple MSI data sets that together show a complex biological process like germination. This might be due to the ongoing increase in spatial and spectral resolution as well as the addition of the third dimension to MSI, which have posed ever-new challenges for the analysis of even a single data set.

However, we believe that even with lower spatial and spectral resolution, MSI shows clear potential to answer many new biological questions addressing the spatio-temporal self-organization in biological systems, and that this is–as is often the case–a simple trade-off between increased resolution in time and reduced resolution in other dimensions.

## Conclusion

This study provides a basis for MSI of cereals with a detailed protocol for sectioning, a comprehensive list of identifications, spatio-temporal information on barley germination as well as the application of unsupervised cluster analyses to assign tissue-specific and time-dependent metabolite patterns for comparative MSI analysis. These results and bioinformatics approaches can be transferred to other MSI datasets. MALDI MSI is capable to simultaneously monitor many important cereal metabolites like lipids, oligosaccharides, and antifungal secondary metabolite during germination. In addition, the specific localization of the metabolites is provided, which is of high advantage over liquid based analyses. This is of high importance for agriculture and crop research: First, the change and localization of lipid profiles are critical aspects for dietary and sensory quality. Second, MSI could visualize ongoing endosperm degradation to aid breeding for optimal germination performance. Third, the detailed localization of antifungal metabolites will certainly support the elucidation of their biological functions, which will be definitely a target to improve natural pathogen resistance to increase yield and quality of the cereals.

## Supporting Information

S1 Fig Sample preparation for MS imaging of barley seeds. (PDF)

S2 Fig Clustering and color coding. (PDF)

S3 Fig Visualization as cluster map and interactive exploration. (PDF)

S4 Fig Longitudinal and transversal cryo-sections of barley. (PDF)

S5 Fig Localization of sodium [M+Na]^+^ and potassium [M+K]^+^ adducts of oligosaccharides in germinating barley. (PDF)

S6 Fig Localization of the sodium and potassium adducts of oligosaccharides in three days germinated barley and their overlay. (PDF)

S7 Fig Localization of hordatine A, B, and C and their glycosylated forms in barley during germination.
(PDF)

S8 Fig Mean mass spectra of annotated regions of interest on barley seed tissues. (PDF)

S1 Supporting Information FT-ICR MSI and MALDI-TOF MS/MS for the identification of barley compounds. A) Comparison of the average TIC obtained by MALDI-TOF MSI and MALDI-FT-ICR MSI of a longitudinal seed section of germinated barley. B) Peak shape and mass resolution of MALDI-TOF MS and MALDI-FT-ICR MS.
(DOCX)

S2 Supporting Information Identification and localization of lipids. A) MALDI-TOF MS/MS of monoacyl phosphatidylcholine (16:0). B) Detection and localization of phosphatidylcholines in barley during germination.
(DOCX)

S3 Supporting Information Identification of carbohydrates. (DOCX)

S1 Table A) FT-ICR and MALDI-TOF based identification of compounds. B) Peak list of the average mass spectrum from MALDI-FT-ICR MS Imaging of a barley seed section. C) Metlin database search results of the 100 highest peaks in the average mass spectrum of FT-ICR MS Imaging of barley.
(XLSX)

## References

[1] http://faostat3.fao.org/download/Q/QC/E, data export of Crops, World + (Total), Cereals,Total > (List), Production Quantity, 2013; data retrieved on 18/08/2015

[2] Steele K, Dickin E, Keerio M, Samad S, Kambona C, Brook R, et al Breeding low-glycemic index barley for functional food. Field Crops Res. 2013: 31–39. 10.1016/j.fcr.2013.07.018

[3] Izydorczyk M S, McMillan T, Bazin S, Kletke J, Dushnicky L, Dexter J, et al Milling of Canadian oats and barley for functional food ingredients: Oat bran and barley fibre-rich fractions. Can. J. Plant Sci. 2014; 3: 573–586. 10.4141/cjps2013-229

[4] Finnie C, Andersen B, Shahpiri A, Svensson B. Proteomes of the barley aleurone layer: A model system for plant signalling and protein secretion. Proteomics. 2011 10.1002/pmic.201000656 21433287

[5] Narziß L, Back W. Die Technologie der Malzbereitung. 8th ed. Wiley-VCH, Weinheim, 2012.

[6] Finnie C, Svensson B. Barley seed proteomics from spots to structures. J. Proteomics. 2009; 3: 315–324. 10.1016/j.jprot.2008.12.001 19118654

[7] Sreenivasulu N, Usadel B, Winter A, Radchuk V, Scholz U, Stein N, et al Barley Grain Maturation and Germination: Metabolic Pathway and Regulatory Network Commonalities and Differences Highlighted by New MapMan/PageMan Profiling Tools. Plant Phys. 2008; 4: 1738–1758. 10.1104/pp.107.111781 PMC2287347 18281415

[8] Gorzolka K, Lissel M, Kessler N, Loch-Ahring S, Niehaus K. Metabolite fingerprinting of barley whole seeds, endosperms, and embryos during industrial malting. J. Biotechnol. 2012; 3: 177–187. 10.1016/j.jbiotec.2012.03.012 22465293

[9] Lee YJ, Perdian DC, Song Z, Yeung ES, Nikolau BJ. Use of mass spectrometry for imaging metabolites in plants. Plant J. 2012; 1: 81–95. 10.1111/j.1365-313X.2012.04899.x 22449044

[10] Römpp A, Spengler B. Mass spectrometry imaging with high resolution in mass and space. Histochem. Cell Biol. 2013; 6: 759–783. 10.1007/s00418-013-1097-6 PMC3656243 23652571

[11] Spengler B. Mass Spectrometry Imaging of Biomolecular Information. Anal. Chem. 2015; 1: 64–82. 10.1021/ac504543v 25490190

[12] Bjarnholt N, Li B, D'Alvise J, Janfelt C. Mass spectrometry imaging of plant metabolites–principles and possibilities. Nat. Prod. Rep. 2014; 6: 818–837. 10.1039/c3np70100j 24452137

[13] Boughton B A, Thinagaran D, Sarabia D, Bacic A, Roessner U. Mass spectrometry imaging for plant biology: a review. Phytochem Rev. 2015 10.1007/s11101-015-9440-2 PMC4870303 27340381

[14] Prideaux B, Stoeckli M. Mass spectrometry imaging for drug distribution studies. J. Proteomics. 2012; 16: 4999–5013. 10.1016/j.jprot.2012.07.028 22842290

[15] Gorzolka K, Bednarz H, Niehaus K. Detection and localization of novel hordatine-like compounds and glycosylated derivates of hordatines by imaging mass spectrometry of barley seeds. Planta. 2014 10.1007/s00425-014-2061-y 24671626

[16] McDonnell LA, Corthals GL, Willems SM, van Remoortere A, van Zeijl RJ, Deelder AM. Peptide and protein imaging mass spectrometry in cancer research. J. Proteomics. 2010; 10: 1921–1944. 10.1016/j.jprot.2010.05.007 20510389

[17] Schöne C, Höfler H, Walch A. MALDI imaging mass spectrometry in cancer research: Combining proteomic profiling and histological evaluation. Clin. Biochem. 2013; 6: 539–545. 10.1016/j.clinbiochem.2013.01.018 23388677

[18] Schwamborn K, Caprioli R M. MALDI Imaging Mass Spectrometry–Painting Molecular Pictures. Mol. Oncol. 2010; 6: 529–538. 10.1016/j.molonc.2010.09.002 PMC2981679 20965799

[19] Matros A, Mock H-P. Mass Spectrometry Based Imaging Techniques for Spatially Resolved Analysis of Molecules. Front. Plant Sci. 2013 10.3389/fpls.2013.00089 PMC3630297 23626593

[20] Zaima N, Goto-Inoue N, Hayasaka T, Setou M. Application of imaging mass spectrometry for the analysis of Oryza sativa rice. Rapid Commun. Mass Spectrom. 2010; 18: 2723–2729. 10.1002/rcm.4693 20814978

[21] Zaima N, Yoshimura Y, Kawamura Y, Moriyama T. Distribution of lysophosphatidylcholine in the endosperm of Oryza sativa rice. Rapid Commun. Mass Spectrom. 2014; 13: 1515–1520. 10.1002/rcm.6927 24861602

[22] Velickovi D, Ropartz D, Guillon F, Saulnier L, Rogniaux H. New insights into the structural and spatial variability of cell-wall polysaccharides during wheat grain development, as revealed through MALDI mass spectrometry imaging. J. Exp. Bot. 2014 10.1093/jxb/eru065 PMC3991742 24600018

[23] Yoshimura Y, Zaima N, Moriyama T, Kawamura Y. Different localization patterns of anthocyanin species in the pericarp of black rice revealed by imaging mass spectrometry. PLoS ONE. 2012; 2: e31285 10.1371/journal.pone.0031285 PMC3281930 22363605

[24] Peukert M, Matros A, Lattanzio G, Kaspar S, Abadía J, Mock H P. Spatially resolved analysis of small molecules by matrix-assisted laser desorption/ionization mass spectrometric imaging (MALDI-MSI). New Phytol. 2012; 193 (3): 806–815. 10.1111/j.1469-8137.2011.03970.x 22126099

[25] Fonville JM, Carter C, Cloarec O, Nicholson JK, Lindon JC, Bunch J, et al Robust data processing and normalization strategy for MALDI mass spectrometric imaging. Anal. Chem. 2012;84(3):1310–1319. 10.1021/ac201767g 22148759

[26] Alexandrov T. MALDI imaging mass spectrometry: statistical data analysis and current computational challenges. BMC bioinformatics. 2012;13(Suppl 16):S11 10.1186/1471-2105-13-S16-S11 23176142 PMC3489526

[27] Rocke DM, Lorenzato S. A two-component model for measurement error in analytical chemistry. Technometrics. 1995;37(2):176–184.

[28] Gibb S, Strimmer K. MALDIquant: a versatile R package for the analysis of mass spectrometry data. Bioinformatics. 2012;28(17):2270–2271. 10.1093/bioinformatics/bts447 22796955

[29] Bemis KD, Harry A, Eberlin LS, Ferreira C, van de Ven SM, Mallick P, et al Cardinal: an R package for statistical analysis of mass spectrometry-based imaging experiments. Bioinformatics. 2015; 31(14):2418–20. 10.1093/bioinformatics/btv146 25777525 PMC4495298

[30] Addie RD, Balluff B, Bovée JV, Morreau H, McDonnell LA. Current State and Future Challenges of Mass Spectrometry Imaging for Clinical Research. Anal. Chem. 2015;. 10.1021/acs.analchem.5b00416 25803124

[31] Palmer AD, Alexandrov T. Serial 3D imaging mass spectrometry at its tipping point. Anal. Chem. 2015;87(8):4055–4062. 10.1021/ac504604g 25817912

[32] Oetjen J, Veselkov K, Watrous J, McKenzie JS, Becker M, Hauberg-Lotte L, et al Benchmark datasets for 3D MALDI-and DESI-imaging mass spectrometry. GigaScience. 2015;4(1):1–8. 25941567 10.1186/s13742-015-0059-4 PMC4418095

[33] Carreira RJ, Shyti R, Balluff B, Abdelmoula WM, van Heiningen SH, van Zeijl RJ, et al Large-Scale Mass Spectrometry Imaging Investigation of Consequences of Cortical Spreading Depression in a Transgenic Mouse Model of Migraine. J. Am. Soc. Mass. Spectrom. 2015;26(6):853–861. 10.1007/s13361-015-1136-8 25877011 PMC4422864

[34] Abdelmoula WM, Carreira RJ, Shyti R, Balluff B, van Zeijl RJ, Tolner EA, et al Automatic registration of mass spectrometry imaging data sets to the Allen brain atlas. Anal. Chem. 2014;86(8):3947–3954. 10.1021/ac500148a 24661141

[35] Leek JT, Scharpf RB, Bravo HC, Simcha D, Langmead B, Johnson WE, et al Tackling the widespread and critical impact of batch effects in high- throughput data. Nat. Rev. Genet. 2010;11(10):733–739. 10.1038/nrg2825 20838408 PMC3880143

[36] Fonville JM, Carter CL, Pizarro L, Steven RT, Palmer AD, Griffiths RL, et al Hyperspectral visualization of mass spectrometry imaging data. Anal. Chem. 2013;85(3):1415–1423. 10.1021/ac302330a 23249247

[37] Palmer AD, Bunch J, Styles IB. The Use of Random Projections for the Analysis of Mass Spectrometry Imaging Data. J. Am. Soc. Mass Spectrom. 2015;26(2):315–322. 10.1007/s13361-014-1024-7 25522725 PMC4320302

[38] Alexandrov T, Becker M, Deininger SO, Ernst G, Wehder L, Grasmair M, et al Spatial segmentation of imaging mass spectrometry data with edge- preserving image denoising and clustering. J. Prot. Res. 2010;9(12):6535–6546. 10.1021/pr100734z 20954702

[39] Alexandrov T, Kobarg JH. Efficient spatial segmentation of large imaging mass spectrometry datasets with spatially aware clustering. Bioinformatics. 2011;27(13):i230–i238. 10.1093/bioinformatics/btr246 21685075 PMC3117346

[40] Strohalm M, Kavan D, Novák P, Volný M, Havlíček V. mMass 3: A Cross-Platform Software Environment for Precise Analysis of Mass Spectrometric Data. Anal. Chem. 2010; 11: 4648–4651. 10.1021/ac100818g 20465224

[41] Smith CA, O'Maille G, Want EJ, Qin C, Trauger SA, Brandon TR, et al METLIN: a metabolite mass spectral database. Ther. Drug Monit. 2005; 6: 747–751. 10.1097/01.ftd.0000179845.53213.39 16404815

[42] Van Der Walt S, Colbert SC, Varoquaux G. The NumPy array: a structure for efficient numerical computation. Comput. Sci. Eng. 2011;13(2):22–30.2

[43] Jones E, Oliphant T, Peterson P, et al SciPy: Open source scientific tools for Python; 2001–. Available from: http://www.scipy.org/.

[44] McKinney W. Data structures for statistical computing in Python. In: Proceedings of the 9th. Phyton in Science Conference. vol. 445; 2010. p. 51–56.

[45] Pedregosa F, Varoquaux G, Gramfort A, Michel V, Thirion B, Grisel O, et al Scikit-learn: Machine Learning in Python. J. Mach. Learn. Res. 2011;12:2825–2830.

[46] Kölling J, Langenkämper D, Abouna S, Khan M, Nattkemper TW. WHIDE A web tool for visual data mining colocation patterns in multivariate bioim- ages. Bioinformatics. 2012;28(8):1143–1150. 10.1093/bioinformatics/bts104 22390938 PMC3324520

[47] Raza SE, Langenkämper D, Epstein DBA, Nattkemper TW, Rajpoot NM. Robust Normalization Protocols for Multiplexed Fluorescence Bioimage Analysis. BioData Min., 2016; in press. 10.1186/s13040-016-0088-2 PMC4779207 26949415

[48] Tennekes M, de Jonge E. Tree colors: color schemes for tree-structured data. Visualization and Computer Graphics, IEEE Transactions on. 2014;20(12):2072–2081. 10.1109/TVCG.2014.2346277 26356921

[49] Ware C. Information visualization: perception for design Elsevier; 2012.

[50] Sumner LW, Amberg A, Barrett D, Beale MH, Beger R, Daykin CA, et al Proposed minimum reporting standards for chemical analysis. Metabolomics. 2007; 3: 211–221. 10.1007/s11306-007-0082-2 24039616 PMC3772505

[51] Price and Parsons. Distribution of lipids in embryonic axis, bran-endosperm, and hull fractions of hulless barley and hulless oat grain. J. Agric. Food Chem., 1979, 27 (4), pp 813–815,

[52] Newman RK, Newman CW. Barley for food and health, Wiley; 2008; 10.1002/9780470369333

[53] Aalenf R B, Opsahl-Ferstad H-G, Linnestad C, Olsen O-A. Transcripts encoding an oleosin and a dormancy-related protein are present in both the aleurone layer and the embryo of developing barley (Hordeum vulgare L.) seeds. Plant J. 1994; 3: 385–396] 10.1111/j.1365-313x.1994.00385.x 8180622

[54] Sugiura Y, Konishi Y, Zaima N, Kajihara S, Nakanishi H, Taguchi R, et al Visualization of the cell-selective distribution of PUFA-containing phosphatidylcholines in mouse brain by imaging mass spectrometry. J. Lipid Res. 2009; 9: 1776–1788. 10.1194/jlr.M900047-JLR200 PMC2724791 19417221

[55] Pihlava J-M. Identification of hordatines and other phenolamides in barley (Hordeum vulgare) and beer by UPLC-QTOF-MS. J. Cereal Sci. 2014; 3: 645–652. 10.1016/j.jcs.2014.07.002

[56] Angel PM, Caprioli RM. Matrix-Assisted Laser Desorption Ionization Imaging Mass Spectrometry: In Situ Molecular Mapping. Biochemistry. 2013; 52(22):3818–28. 10.1021/bi301519p 23259809 PMC3864574

[57] Zaima N, Yoshioka S, Sato Y, Shinano S, Ikeda Y, Moriyama T. Enhanced specificity for phosphatidylcholine analysis by positive ion mode matrix-assisted laser desorption/ionization imaging mass spectrometry. Rapid Commun. Mass Spectrom. 2014; 13: 1453–1458. 10.1002/rcm.6917 24861594

[58] An Y-Q, Lin L. Transcriptional regulatory programs underlying barley germination and regulatory functions of Gibberellin and abscisic acid. BMC Plant Biol. 2011; 1: 105 10.1186/1471-2229-11-105 PMC3130657 21668981

[59] Kubo A, Kajimura M, Suematsu M. Matrix-Assisted Laser Desorption/Ionization (MALDI) Imaging Mass Spectrometry (IMS): A Challenge for Reliable Quantitative Analyses. Mass Spectrom. (Tokyo). 2012; 1: A0004 10.5702/massspectrometry.A0004 24349905 PMC3775825

[60] Howell KA, Narsai R, Carroll A, Ivanova A, Lohse M, Usadel B, et al Mapping Metabolic and Transcript Temporal Switches during Germination in Rice Highlights Specific Transcription Factors and the Role of RNA Instability in the Germination Process. Plant Physiol. 2008; 2: 961–980. 10.1104/pp.108.129874 PMC2633829 19074628

[61] Burhenne K. A New Class of N-Hydroxycinnamoyltransferases. Purification, cloning, and expression of a barley agmatine coumaroyltransferase (EC 2.3.1.64). J. Biol. Chem. 2003; 16: 13919–13927. 10.1074/jbc.M213041200 12582168

[62] Stoessl A, Unwin CH. The antifungal factors in barley. V. Antifungal activity of the hordatines. Can. J. Bot. 1970; 3: 465–470. 10.1139/b70-066

[63] Kageyama N. Elucidation of Chemical Structures of Components Responsible for Beer Aftertaste. ASBC. 2011 10.1094/ASBCJ-2011-0901-01

[64] Smith TA, Best GR. Distribution of the hordatines in barley. Phytochemistry. 1978; 7: 1093–1098. 10.1016/S0031-9422(00)94295-X

[65] Muroi A, Ishihara A, Tanaka C, Ishizuka A, Takabayashi J, Miyoshi H, et al Accumulation of hydroxycinnamic acid amides induced by pathogen infection and identification of agmatine coumaroyltransferase in Arabidopsis thaliana. Planta. 2009; 3: 517–527. 10.1007/s00425-009-0960-0 19521717

[66] Chong E S, McGhie TK, Heyes JA, Stowell KM. Metabolite profiling and quantification of phytochemicals in potato extracts using ultra-high-performance liquid chromatography-mass spectrometry. J. Sci. Food Agric. 2013; 15: 3801–3808. 10.1002/jsfa.6285 23794415

[67] Facchini P J, Hagel J, Zulak K G. Hydroxycinnamic acid amide metabolism: physiology and biochemistry. Can. J. Bot. 2002; 6: 577–589. 10.1139/b02-065

[68] Muroi A, Matsui K, Shimoda T, Kihara H, Ozawa R, Ishihara A, et al Acquired immunity of transgenic torenia plants overexpressing agmatine coumaroyltransferase to pathogens and herbivore pests. Sci. Rep. 2012 10.1038/srep00689 PMC3449287 23008754

[69] Ogura Y, Ishihara A, Iwamura H. Induction of hydroxycinnamic acid amides and tryptophan by jasmonic acid, abscisic acid and osmotic stress in barley leaves. Z Naturforsch C. 2001; 3–4: 193–202. 10.1515/znc-2001-3-405 11371008

[70] von Ropenack E, Parr A, Schulze-Lefert P. Structural Analyses and Dynamics of Soluble and Cell Wall-bound Phenolics in a Broad Spectrum Resistance to the Powdery Mildew Fungus in Barley. J. Biol. Chem. 1998; 15: 9013–9022. 10.1074/jbc.273.15.9013 9535889

[71] Palmer A, Ovchinnikova E, Thuné M, Lavigne R, Guével B, Dyatlov A, et al Using collective expert judgements to evaluate quality measures of mass spectrometry images. Bioinformatics. 2015;31(12):i375–i384. 10.1093/bioinformatics/btv266 26072506 PMC4765867

