# Supplementary material for: Spatio-Temporal Metabolite Profiling of the Barley Germination Process by MALDI MS Imaging
Source: PLoS One. 2016 Mar 3;11(3):e0150208. doi: 10.1371/journal.pone.0150208 (PMC4777520; doi:10.1371/journal.pone.0150208)
Supplement: S1 Fig — (PDF) [file pone.0150208.s001.pdf]

S1 Fig: Sample preparation for MS imaging of barley seeds

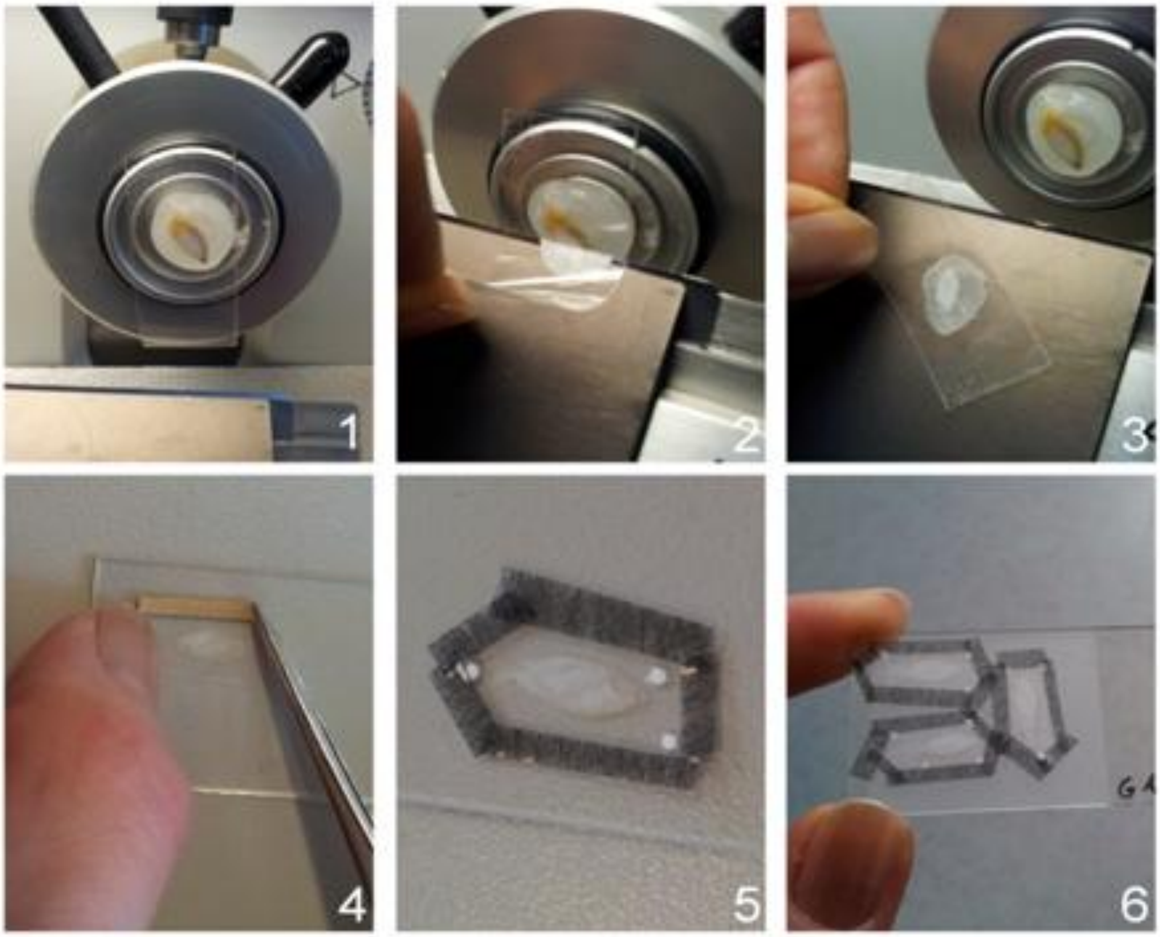

S1 Fig: Overview on the sample preparation for MSI of barley.

1. A barley seed was embedded in ice. Half of the seed was cut away and adhesive film was stuck on the seed.
2. The seed section was stabilized by the film during cutting.
3. The section on the adhesive film (upwards) was directly transferred to a vacuum desiccator.
4. After vacuum drying, the section was mounted with conductive tape (black) on an indium tin oxide (ITO) coated glass slide.
5. The mounted seed section with teaching points (white).
6. Samples after matrix application, ready for MS imaging.
