# Supplementary material for: Spatio-Temporal Metabolite Profiling of the Barley Germination Process by MALDI MS Imaging
Source: PLoS One. 2016 Mar 3;11(3):e0150208. doi: 10.1371/journal.pone.0150208 (PMC4777520; doi:10.1371/journal.pone.0150208)
Supplement: S2 Fig — (PDF) [file pone.0150208.s002.pdf]

S2 Fig: Clustering and color coding

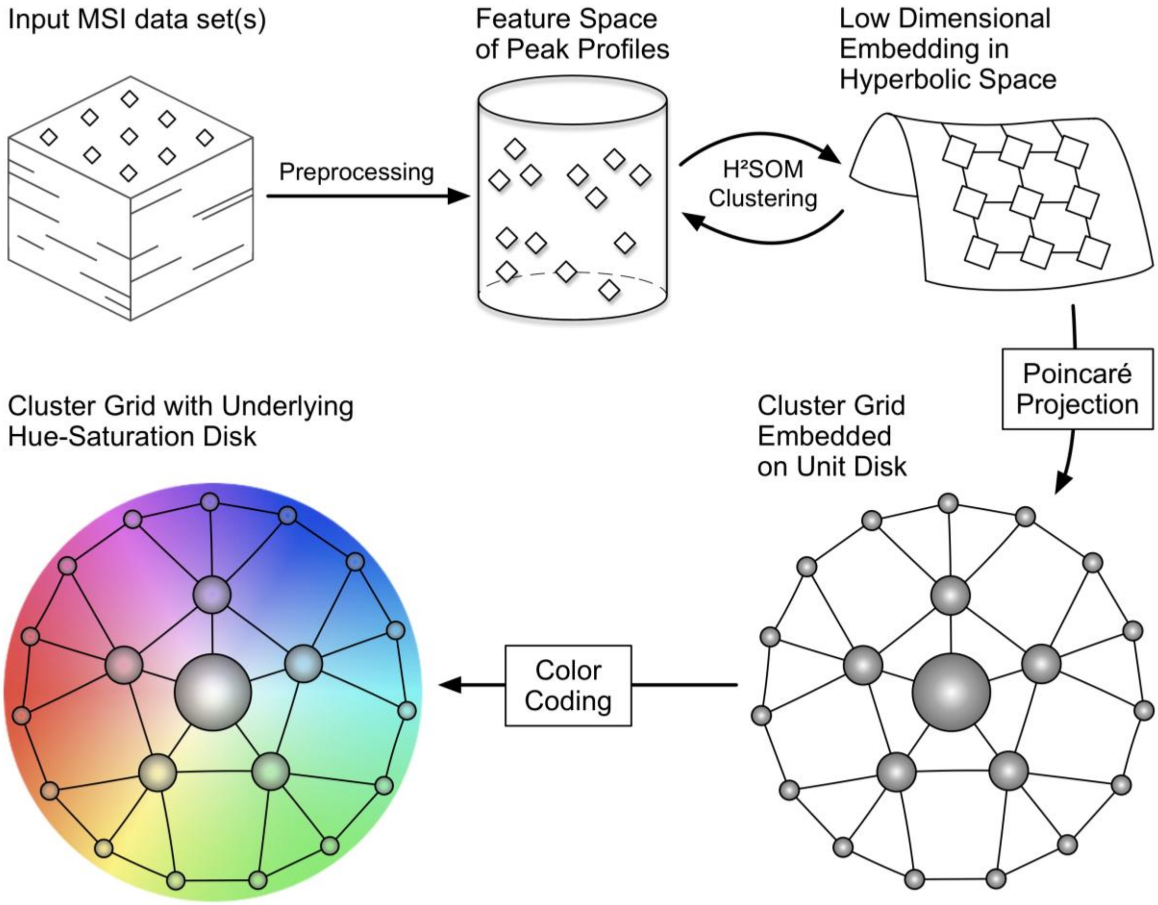

S2 Fig: Clustering and color coding. The MSI data sets are preprocessed and the derived peak profiles build up the feature space for the clustering. The H^2SOM algorithm learns a low dimensional embedding of a grid of clusters of the data in hyperbolic space. To use the properties of this embedding for visualization, the Poincaré disk model is used to project the grid of cluster nodes on a unit disk. This disk is then overlaid on a hue-saturation disk in the user interface and each node is colored by the now underlying color. The resulting color code is used to visualize the clustering in the context of the original data set and enable interactive exploration (see S3 Fig).
