# Supplementary material for: Spatio-Temporal Metabolite Profiling of the Barley Germination Process by MALDI MS Imaging
Source: PLoS One. 2016 Mar 3;11(3):e0150208. doi: 10.1371/journal.pone.0150208 (PMC4777520; doi:10.1371/journal.pone.0150208)
Supplement: S3 Fig — (PDF) [file pone.0150208.s003.pdf]

S3 Fig: Visualization as cluster map and interactive exploration

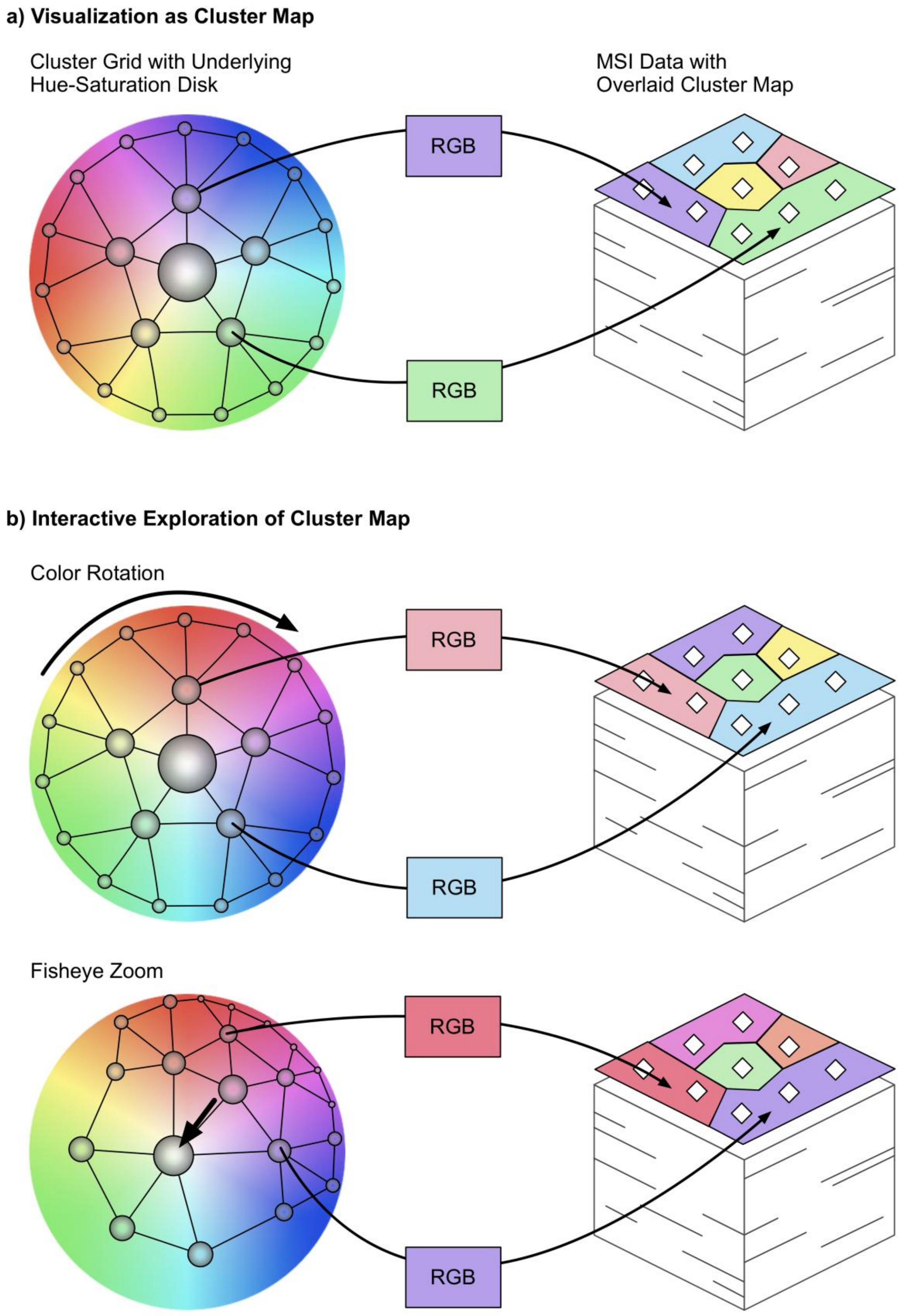

S3 Fig: Visualization as cluster map and interactive exploration. Overview of visualization as cluster map (a) and interactive exploration of cluster map (b). See https://ani.cebitec.uni-bielefeld.de/barleymsi/ for interactive examples.
