# Supplementary material for: Spatio-Temporal Metabolite Profiling of the Barley Germination Process by MALDI MS Imaging
Source: PLoS One. 2016 Mar 3;11(3):e0150208. doi: 10.1371/journal.pone.0150208 (PMC4777520; doi:10.1371/journal.pone.0150208)
Supplement: S4 Fig — (PDF) [file pone.0150208.s004.pdf]

S4 Fig: Longitudinal and transversal cryo-sections of barley

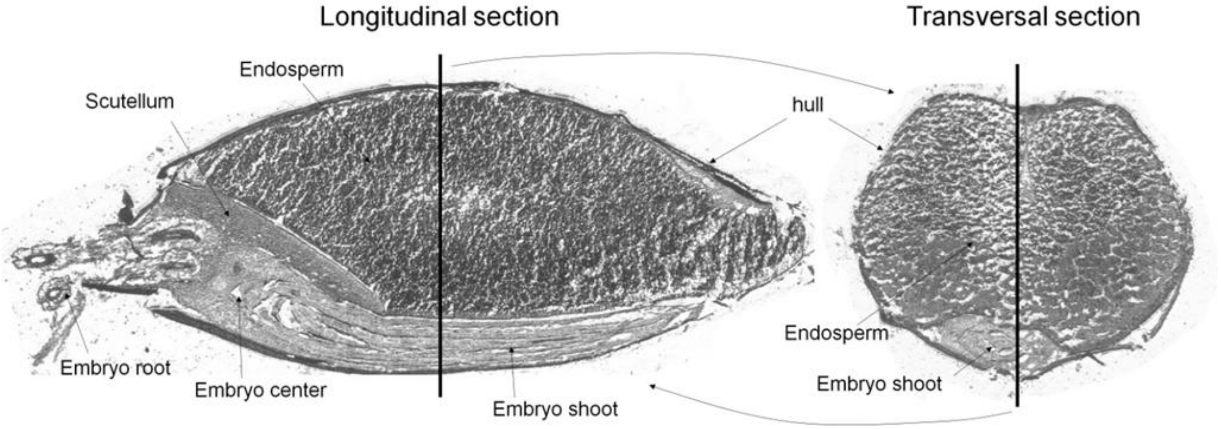

S4 Fig: Longitudinal and transversal cryo-sections of a barley seed at the third day of germination. Main seed organs and tissues are indicated; the transversal section was at half of the total seed length in all samples, the longitudinal section in the middle of the seed through the embryo axis (black lines).
