# Supplementary material for: Spatio-Temporal Metabolite Profiling of the Barley Germination Process by MALDI MS Imaging
Source: PLoS One. 2016 Mar 3;11(3):e0150208. doi: 10.1371/journal.pone.0150208 (PMC4777520; doi:10.1371/journal.pone.0150208)
Supplement: S5 Fig — (PDF) [file pone.0150208.s005.pdf]

S5 Fig: Localization of sodium $[M+Na]^+$ and potassium $[M+K]^+$ adducts of oligosaccharides in germinating barley

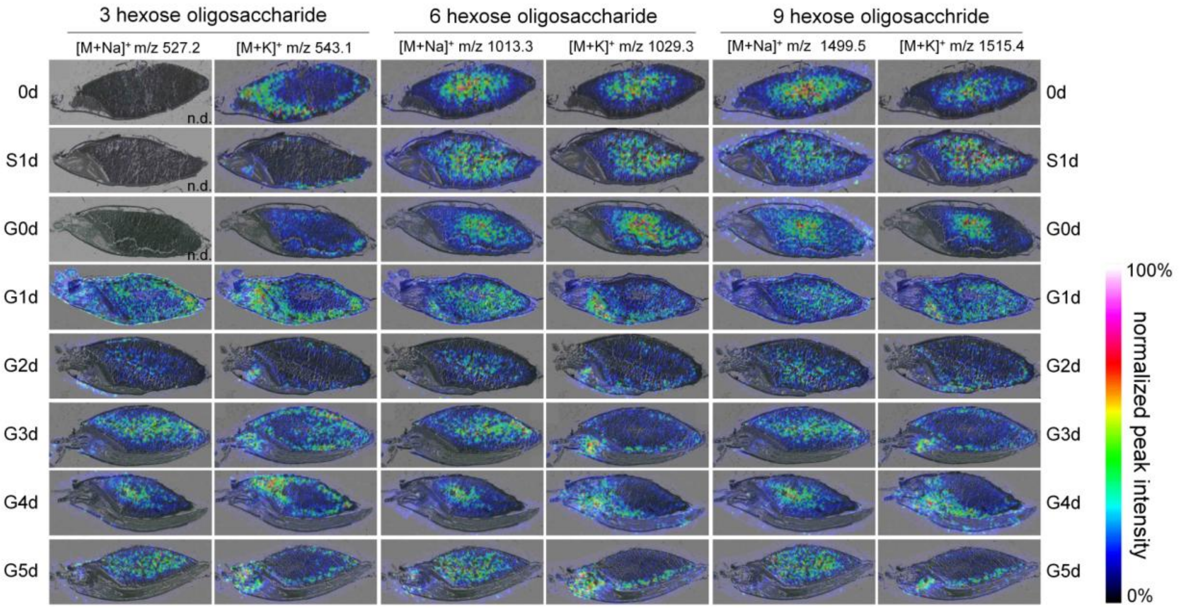

S5 Fig: Localization of sodium $[M+Na]^+$ and potassium $[M+H]^+$ adducts of oligosaccharides with three, six, and nine hexoses in barley during germination. MS intensities were normalized to the TIC of each mass spectrum, the highest relative intensity of all MS was set to 100% (see legend). 0d: barley, S1d: steeped barley, G0d–G5d: days of germination; see Fig 1. n.d.: not detected (*m/z* 527.2 in 0d, S1d, and G0d samples).
