# Supplementary material for: Spatio-Temporal Metabolite Profiling of the Barley Germination Process by MALDI MS Imaging
Source: PLoS One. 2016 Mar 3;11(3):e0150208. doi: 10.1371/journal.pone.0150208 (PMC4777520; doi:10.1371/journal.pone.0150208)
Supplement: S6 Fig — (PDF) [file pone.0150208.s006.pdf]

S6 Fig: Localization of the sodium and potassium adducts of oligosaccharides in three days germinated barley and their overlay

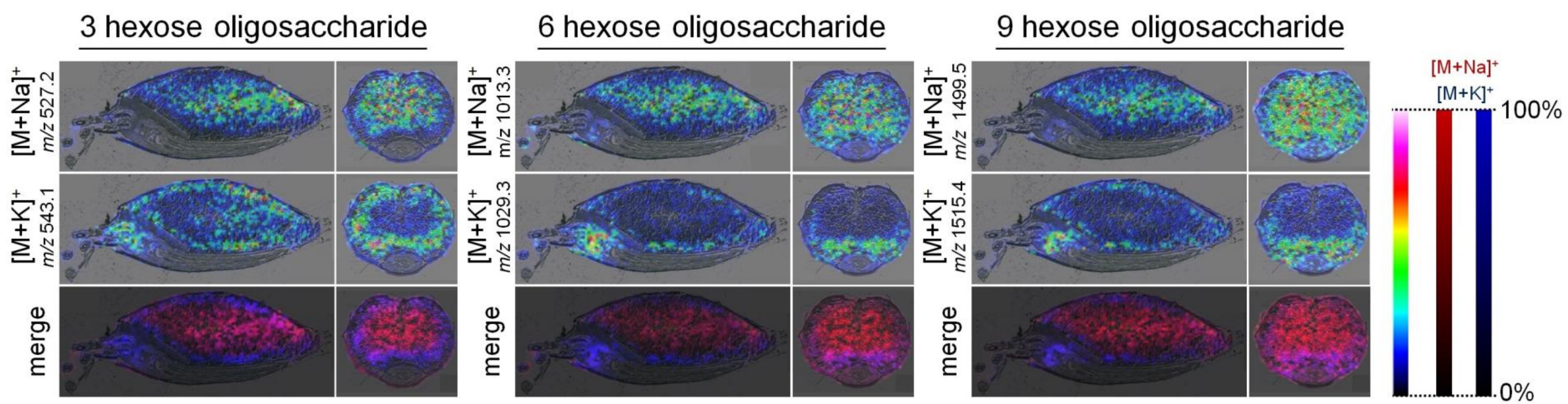

S6 Fig: Localization of the sodium and potassium adduct oligosaccharides in three day germinated barley in longitudinal and transversal sections. Oligomers of three, six, and nine hexoses length were chosen as examples with peak intensities displayed in rainbow color code. In the merge of the *m/z* channels, the sodium adduct is displayed in red and the potassium adduct in blue. MS intensities were normalized to the TIC of each mass spectrum, the highest relative intensity of all MS was set to 100%.
