# Supplementary material for: Spatio-Temporal Metabolite Profiling of the Barley Germination Process by MALDI MS Imaging
Source: PLoS One. 2016 Mar 3;11(3):e0150208. doi: 10.1371/journal.pone.0150208 (PMC4777520; doi:10.1371/journal.pone.0150208)
Supplement: S7 Fig — Localization of hordatine A, B, and C and their glycosylated forms in barley during germination. (PDF) [file pone.0150208.s007.pdf]

S7 Fig: Localization of hordatine A, B, and C and their glycosylated forms in germinating barley

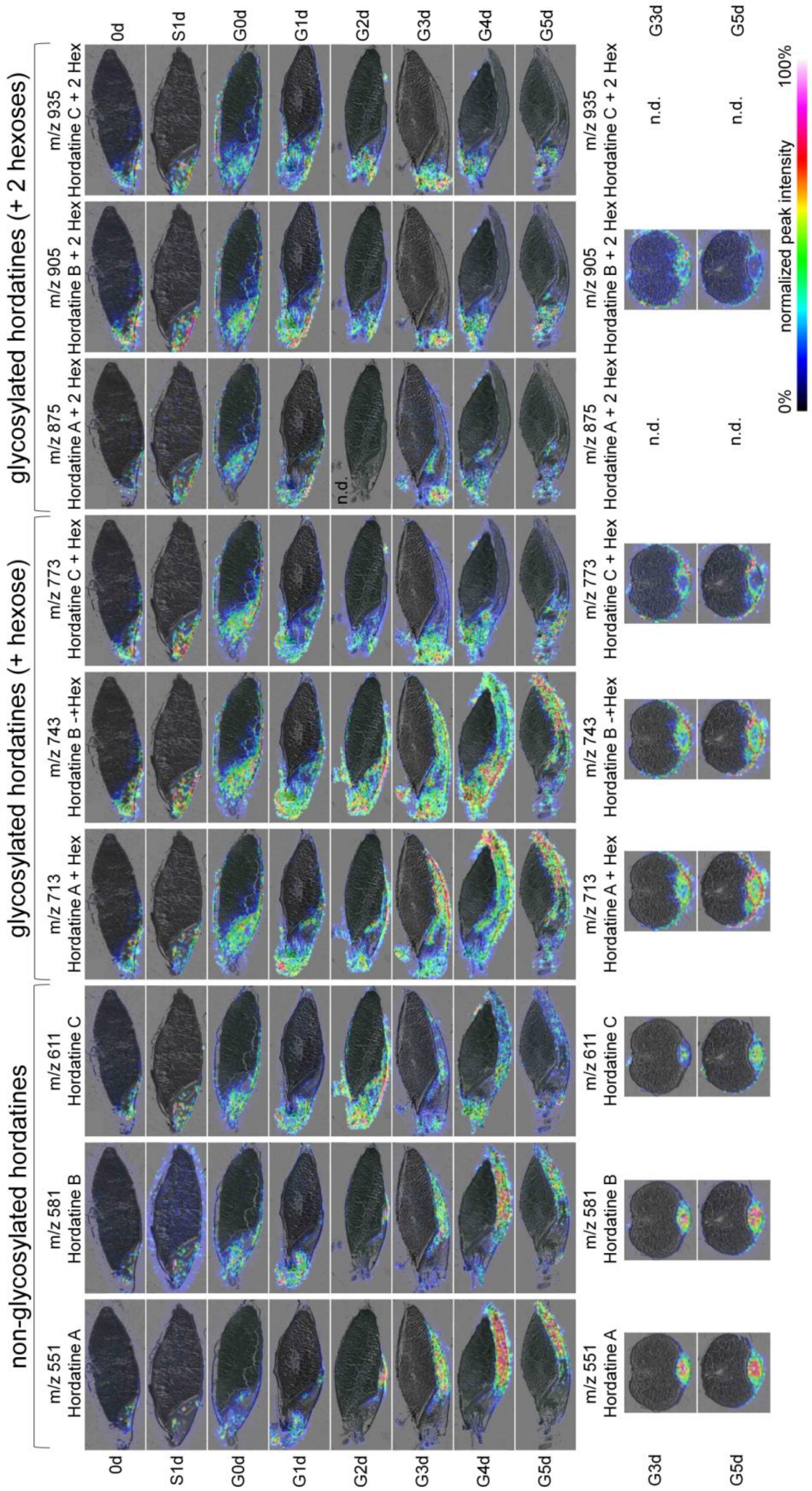

S7 Fig: Localization of hordatine A, B, and C in their non-glycosylated (*m/z* 551, 581, 611), glycosylated (713, 743, 773), and disaccharide-modified forms (875, 905, 935) during germination (0d: ungerminated barley, S1d, steeped barley, G0d–G5d: days germination, see Fig 1). Longitudinal (top) and transversal (bottom) sections are presented. The compounds were not detected in cross sections in ungerminated barley; n.d. (not detected) indicates *m/z* values that were below the detection threshold. Intensities of the selected *m/z* values were normalized to the TIC of each mass spectrum, the highest relative intensity was set to 100%.
