# Supplementary material for: Spatio-Temporal Metabolite Profiling of the Barley Germination Process by MALDI MS Imaging
Source: PLoS One. 2016 Mar 3;11(3):e0150208. doi: 10.1371/journal.pone.0150208 (PMC4777520; doi:10.1371/journal.pone.0150208)
Supplement: S8 Fig — (PDF) [file pone.0150208.s008.pdf]

S8 Fig: Mean mass spectra of annotated regions of interest on barley seed tissues

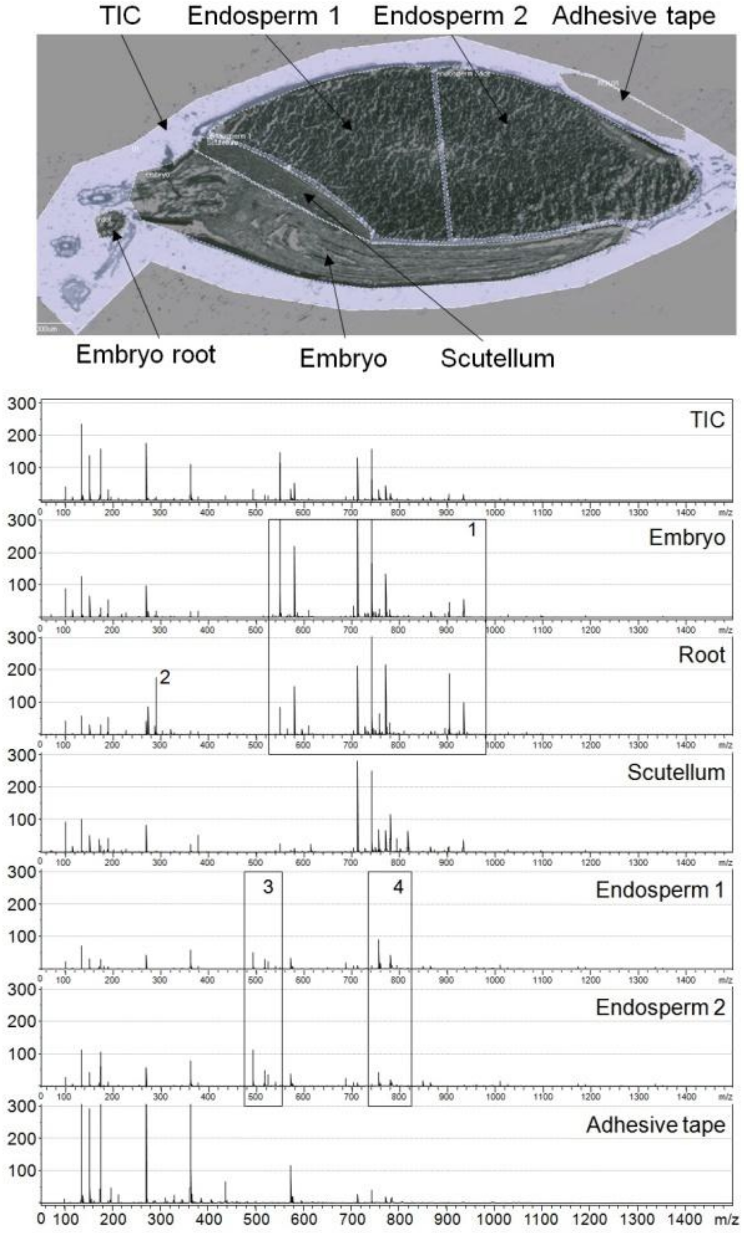

S8 Fig: Mass spectrometric profiles of tissues of a three day germinated barley seed (G3d) from *m/z* 0 to 1500 *m/z*; signals of higher *m/z* values were observed (Table 1), but not displayed in the overview. Top: Regions of interest were assigned with respect to the global sum of all MS (TIC, complete measurement area), the embryo, a root, the scutellum, and the background evaluation with the adhesive tape. The endosperm was separated in two regions (1 and 2). Bottom: Mass spectra display the sum of all MS that were acquired in the assigned sample regions, normalized to the TIC and the number of laser shots. The intensity is displayed in arbitrary units, scaled to the same value. Boxes indicate tissue specific signals with 1: hordatines, 2: coumaroylagmatine, 3: monoacyl phosphatidylcholines, and 4: diacyl phosphatidylcholines.
