# Supplementary material for: Spatio-Temporal Metabolite Profiling of the Barley Germination Process by MALDI MS Imaging
Source: PLoS One. 2016 Mar 3;11(3):e0150208. doi: 10.1371/journal.pone.0150208 (PMC4777520; doi:10.1371/journal.pone.0150208)
Supplement: S1 Supporting Information — A) Comparison of the average TIC obtained by MALDI-TOF MSI and MALDI-FT-ICR MSI of a longitudinal seed section of germinated barley. B) Peak shape and mass resolution of MALDI-TOF MS and MALDI-FT-ICR MS. (DOCX) [file pone.0150208.s009.docx]

**S1 Supporting Information: FT-ICR MSI and MALDI-TOF MS/MS for the identification of barley compounds**

**A) Comparison of the average TIC obtained by MALDI-TOF MSI and MALDI-FT-ICR MSI of a longitudinal seed section of germinated barley**


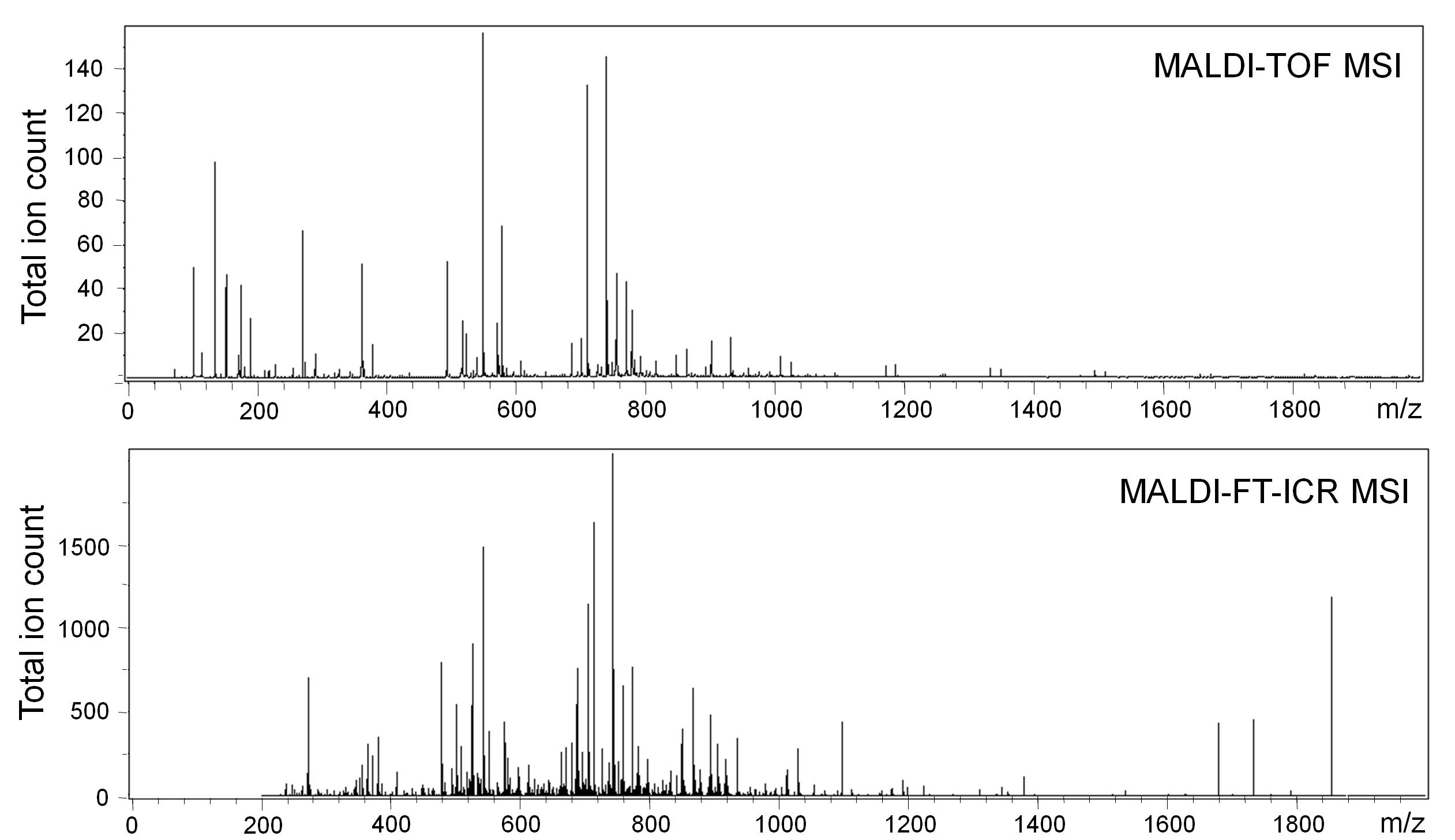


**S1 Supporting Information Figure A:** Comparison of the average mass spectra revealed from MALDI MS Imaging of barley using Time Of Flight (TOF) MS or Fourier Transform Ion Cyclotron Resonance (FT-ICR) mass spectrometry. “No normalization” was selected for both overlay spectra. *m/z* 0 – 2000 is displayed in the comparison; ions were collected from *m/z* 0 – 3000 (TOF) and *m/z* 200 – 2000 (FT-ICR) in positive ion mode.

**B) Peak shape and mass resolution of MALDI-TOF MS and MALDI-FT-ICR MS.**


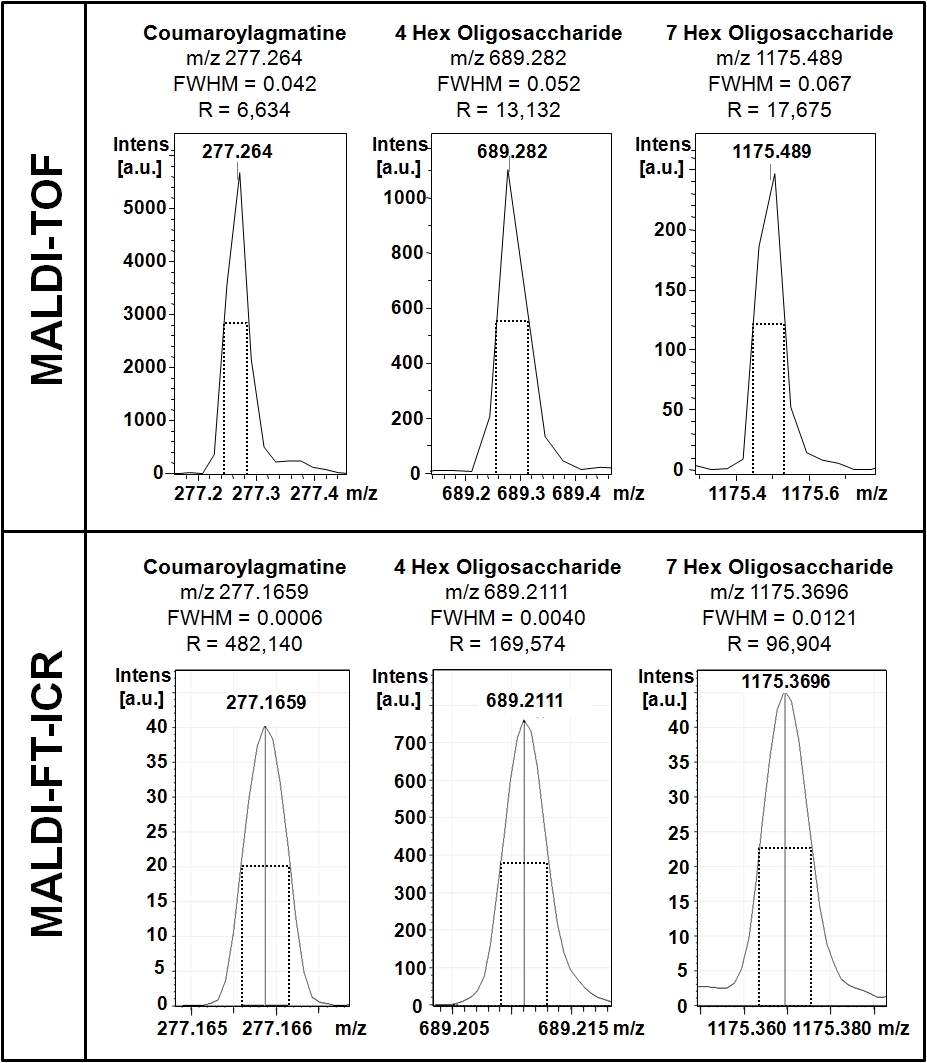


**S1 Supporting Information Figure B:** Comparison of the peak shape and resolution of mass spectra from MALDI-TOF MS and MALDI-FT-ICR MS. Three representative compounds were selected (coumaroylagmatine, 4 Hex Oligosaccharide: oligomer of 4 hexose residues [M+Na]^+^, 7 Hex Oligosaccharide: oligomer of 4 hexose residues [M+Na]^+^) to underline the connection between the *m/z*, the FWHM (full width at half maximum) and the resulting mass resolution (R = *m/z* / FWHM): The larger the *m/z*, the wider the peak and the lower the mass resolution. Intensities are provided as arbitrary units [a.u.].

Automated peak picking in FlexAnalysis (Bruker Daltonics) on MALDI-TOF MS revealed peak resolutions between R=5000 and R=20000 with a mean of R=10170, calculated from several representative mass spectra. The FWHM ranged between 0.016 and 0.116 with a mean of 0.049. MALDI-FT-ICR MS revealed peak resolution values between R=4000 (low intense peaks) and R=1200000 (at low masses), the mean was R=113500 as calculated from the overlay TIC. The FWHM ranged between 0.0002 at low masses and 0.015 at high masses.
