# Supplementary material for: Spatio-Temporal Metabolite Profiling of the Barley Germination Process by MALDI MS Imaging
Source: PLoS One. 2016 Mar 3;11(3):e0150208. doi: 10.1371/journal.pone.0150208 (PMC4777520; doi:10.1371/journal.pone.0150208)
Supplement: S2 Supporting Information — A) MALDI-TOF MS/MS of monoacyl phosphatidylcholine (16:0). B) Detection and localization of phosphatidylcholines in barley during germination. (DOCX) [file pone.0150208.s010.docx]

**S2 Supporting Information: Identification and localization of lipids**

The *Metlin* database identified phosphatidylcholines (PC), phosphatidylinositols (PI), phosphatidylethanolamines (PE), and triglycerides with several possible fatty acid compositions for each *m/z* value (see S1 Table C). MS/MS could not resolve the exact fatty acid structures, but validated the identifications of all PCs by the detection of the characteristic head group as exemplary shown in for PC (16:0). The MS/MS of the proposed phosphatidylinositols and triglycerides were not sufficient to refuse or to validate their structures due to low precursor intensity or isobaric secondary metabolites.

**A) MALDI-TOF MS/MS of monoacyl phosphatidylcholine (16:0)**


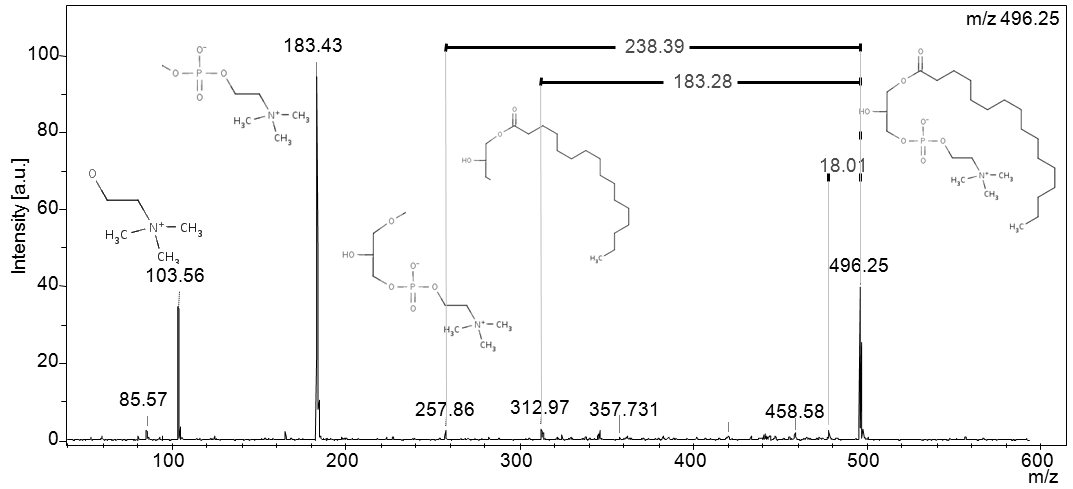


**S2 Supporting Information Figure A:** MALDI-TOF MS/MS of *m/z* 496, identified as monoacyl phosphatidylcholine (16:0) [M+H]^+^, see Table 1. MS/MS was obtained from the endosperm of a sectioned matrix-coated barley seed and processed in FlexAnalysis (Bruker Daltonics). Molecules of a reference spectrum from the *Metlin* database were assigned to peaks. Mass differences are shown with horizontal bars [Da].

PCs with one and two fatty acids demonstrated distinct localizations. All monoacyl PCs were detected in the endosperm – preferably in the distal parts – during the whole germination process. All diacyl PCs were observed in the aleurone layer and the embryo in ungerminated barley. After imbibition and in course of germination, these compounds occurred in the scutellum and in the peripheral endosperm near the husk (see S2 Supporting Information Figure B). This localization highly corresponded to the potassium adducts of the oligosaccharides. However, the different adducts of PCs did not exhibit any significant spatial segregation as described for the oligosaccharides.

**B) Detection and localization of phosphatidylcholines in barley during germination**


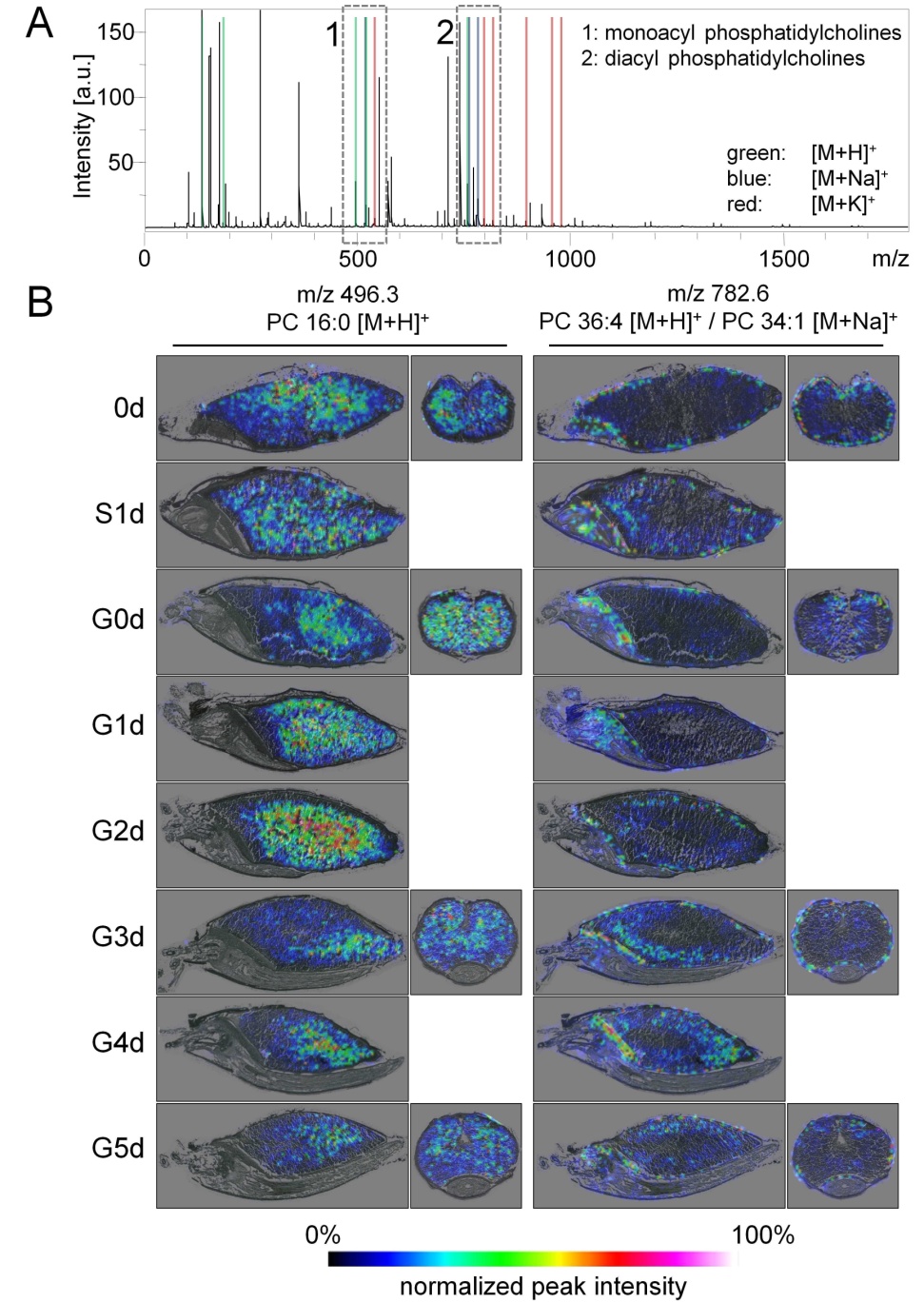


**S2 Supporting Information Figure B:** Detection and localization of representative phosphatidylcholines (PCs) in longitudinal and transversal seed sections of barley during germination. A) Average mass spectrum with annotated lipids detected as [M+H]^+^ (green), [M+Na]^+^ (blue), and [M+K]^+^ (red), see Table 1 and S1 Table A. B) MS intensity heat maps of one biological replicate for each sampling time point during germination. The [M+H]^+^ of PC (16:0) is displayed as representative for monoacyl PC localizations; *m/z* 782.6 is a diacyl PC of either the [M+H]^+^ of PC(36:4) (in total 36 carbon atom length with four double bonds) or PC(34:1) as [M+Na]^+^ (34 carbon atoms in total with one double bond), see Table 1. Intensities of the selected *m/z* values were normalized to the TIC of each mass spectrum, the highest relative intensity was set to 100%. Time points: 0d: barley, S1d: steeped barley, G0d–G5d: days of germination, see Fig 1.
