# Supplementary material for: Spatio-Temporal Metabolite Profiling of the Barley Germination Process by MALDI MS Imaging
Source: PLoS One. 2016 Mar 3;11(3):e0150208. doi: 10.1371/journal.pone.0150208 (PMC4777520; doi:10.1371/journal.pone.0150208)
Supplement: S3 Supporting Information — (DOCX) [file pone.0150208.s011.docx]

**S3 Supporting Information: Identification of carbohydrates**

A very regular peak pattern was observed in the MSI data, which covered a mass range from 381 *m/z* to 2164 *m/z* (Table 1). Several of these peaks retrieved *Metlin* database hits as sodium and potassium adducts of oligosaccharides. The same MS pattern was observed in aqueous barley seed extracts with much higher intensities (S3 Supporting Information Figure A), so crude extracts were chosen for MS/MS analyses. The mass spectra of crude extracts and a Dextrin 20 oligosaccharide standard demonstrated equal peak masses (S3 Supporting Information Figure A). In the MS/MS of selected precursors, mass differences of hexose residues (162.05 Da) were annotated. Double peaks were observed ($\Delta m=18.04 Da$), which derived from the neutral loss of water (S3 Supporting Information Figure A, right). Based on the database results, MS/MS, and the highly regular MS peak pattern, all 22 compounds of this cluster were annotated as sodium and potassium adducts of oligosaccharides with lengths from 2 to 13 hexose residues (Table 1) of which the [M+H]^+^ peak was not detected.

**A) MS and MS/MS of oligosaccharides**


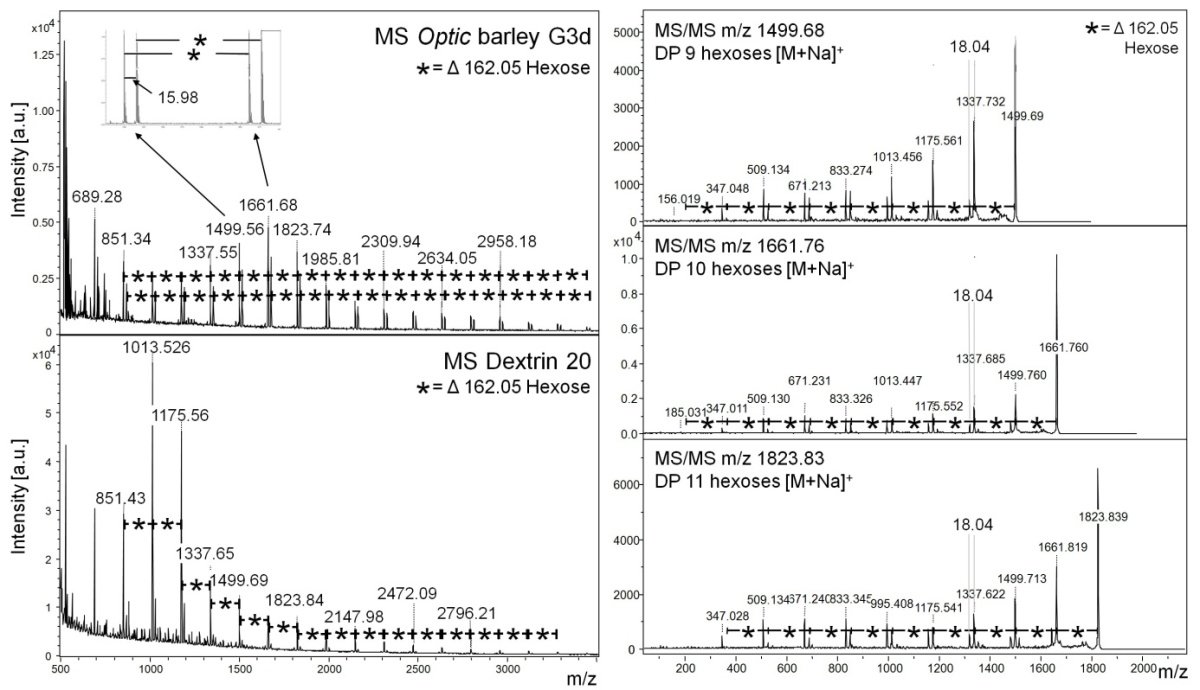


**S3 Supporting Information Figure A:** Mass spectra of aqueous extracts of barley seeds after three days of germination with magnified view on the double peaks (top) and MS of Dextrin extracts (bottom) in the range of 500 to 3500 *m/z*. Left: MS/MS of oligosaccharides of three degrees of polymerization (DP). Mass differences were indicated by bars with the asterisk * replacing 162 Da, a hexose residue. Double peaks in the MS differed by 15.98 Da (distance of sodium and potassium adduct), double peaks in the MS/MS were neutral losses of water (18.04 Da). Signal intensities were normalized to ion counts per laser shot.
